# Facile synthesis of near-infrared responsive on-demand oxygen releasing nanoplatform for precise MRI-guided theranostics of hypoxia-induced tumor chemoresistance and metastasis in triple negative breast cancer

**DOI:** 10.1186/s12951-022-01294-z

**Published:** 2022-03-04

**Authors:** Dong Zhang, Yuanyuan You, Yuan Xu, Qingqing Cheng, Zeyu Xiao, Tianfeng Chen, Changzheng Shi, Liangping Luo

**Affiliations:** 1 Department of Medical Imaging Center, The First Affiliated Hospital, Jinan University, Guangzhou, 510630 China; 2 Zhuhai Precision Medical Center, Guangdong Provincial Key Laboratory of Tumor Interventional Diagnosis and Treatment, Zhuhai People’s Hospital, Zhuhai Hospital Affiliated With Jinan University, Jinan University, Zhuhai, 519000 Guangdong People’s Republic of China; 3 The Shunde Affiliated Hospital, Jinan University, Foshan, 528300 China

**Keywords:** On-demand drug release, MRI-guided theranostics, Tumor hypoxia, Nanoplatform, Triple negative breast cancer

## Abstract

**Background:**

Hypoxia is an important factor that contributes to chemoresistance and metastasis in triple negative breast cancer (TNBC), and alleviating hypoxia microenvironment can enhance the anti-tumor efficacy and also inhibit tumor invasion.

**Methods:**

A near-infrared (NIR) responsive on-demand oxygen releasing nanoplatform (O_2_-PPSiI) was successfully synthesized by a two-stage self-assembly process to overcome the hypoxia-induced tumor chemoresistance and metastasis. We embedded drug-loaded poly (lactic-co-glycolic acid) cores into an ultrathin silica shell attached with paramagnetic Gd-DTPA to develop a Magnetic Resonance Imaging (MRI)-guided NIR-responsive on-demand drug releasing nanosystem, where indocyanine green was used as a photothermal converter to trigger the oxygen and drug release under NIR irradiation.

**Results:**

The near-infrared responsive on-demand oxygen releasing nanoplatform O_2_-PPSiI was chemically synthesized in this study by a two-stage self-assembly process, which could deliver oxygen and release it under NIR irradiation to relieve hypoxia, improving the therapeutic effect of chemotherapy and suppressed tumor metastasis. This smart design achieves the following advantages: (i) the O_2_ in this nanosystem can be precisely released by an NIR-responsive silica shell rupture; (ii) the dynamic biodistribution process of O_2_-PPSiI was monitored in real-time and quantitatively analyzed via sensitive MR imaging of the tumor; (iii) O_2_-PPSiI could alleviate tumor hypoxia by releasing O_2_ within the tumor upon NIR laser excitation; (iv) The migration and invasion abilities of the TNBC tumor were weakened by inhibiting the process of EMT as a result of the synergistic therapy of NIR-triggered O_2_-PPSiI.

**Conclusions:**

Our work proposes a smart tactic guided by MRI and presents a valid approach for the reasonable design of NIR-responsive on-demand drug-releasing nanomedicine systems for precise theranostics in TNBC.

**Graphical Abstract:**

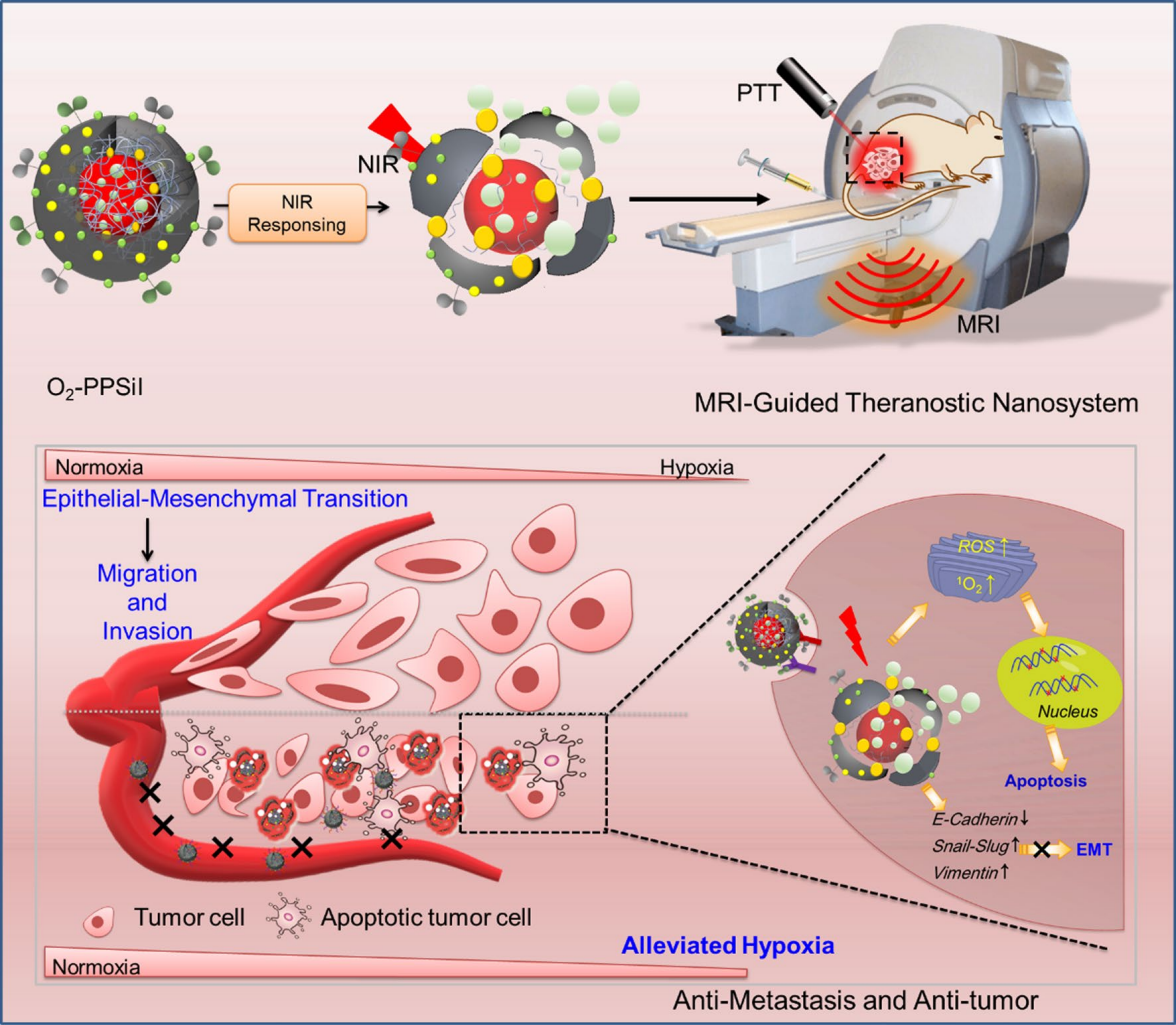

**Supplementary Information:**

The online version contains supplementary material available at 10.1186/s12951-022-01294-z.

## Introduction

Triple-negative breast cancer (TNBC) accounts for almost 15–20% of all the breast cancer cases and presents a poor prognosis due to its metastatic nature and high recurrence rate [1, 2]. Owing to the paucity of ER, PR and HER2, TNBC cannot benefit from the FDA-approved targeted therapies which have been proved to be efficacious for the other breast cancer subtypes [3, 4]. The systemic chemotherapy using paclitaxel (PTx) remains the first-line treatment of advanced TNBC, but its efficacy is limited by its severe side effects and acquired drug resistance, leading to the failure of chemotherapy and tumor migration and invasion [5, 6]. The hypoxic tumor microenvironment was reported to be involved in different tumorigenesis mechanisms of TNBC, such as invasion, immune evasion, chemoresistance, and metastasis [7]. Hypoxia is a pathophysiological characteristic for the tumor microenvironment that results in abnormalities in the tumor vasculature and a disproportion between oxygen provision and oxygen utilization in the tumors [8, 9]. Poor efficacy in hypoxic areas is often linked with the space from cancer cells to supply vessels and the permeability of the newly formed tumor vascular system [10–12]. Meanwhile, hypoxia upregulates the activity of p-glycoprotein in tumor cells, leading to drug resistance of the cells [13]. In addition, most chemotherapeutic agents including PTx induce cytotoxicity by proliferating cells, but hypoxic cancer cells are more likely to proliferate more slowly than normal tissue, resulting in tumor cells tolerant to the chemotherapy. What’s more, hypoxia has increasingly emerged as a crucial factor of microenvironment in the regulation of tumor metastasis accompanying with the activation of hypoxia-inducible transcription factor (HIF), which could activate the process of epithelial-to-mesenchymal transition (EMT), resulting in tumor metastasis and leading to a multidrug resistance [14–16]. Therefore, hypoxia is an important factor that contributes to TNBC metastasis and multidrug resistance to chemotherapy, and alleviating hypoxia microenvironment can enhance the anti-TNBC efficacy of chemotherapy and also inhibit its invasion and metastasis.

Tumor oxygenation can relieve hypoxia to achieve better therapeutic effects [17, 18]. For example, producing oxygen in situ with the use of catalysts to promote the degradation of endogenous hydrogen peroxide (H_2_O_2_) can relieve tumor hypoxia and strengthen the therapy [19–21]. In addition, improving intertumoral blood flow can also lead to relieved tumor hypoxia [22, 23]. However, the limited available H_2_O_2_ within the tumor, as well as the poor distribution of oxidized red blood cells reaching the tumor vessels, often leads to an insufficient intratumor oxygen delivery and unsatisfactory tumor reoxygenation. Biocompatible perfluorocarbons (PFCs) have been applied as the artificial blood substitute for many years, which often been used in different nanocarriers to transport oxygen into tumors for alleviating the tumor hypoxia [24–26]. However, O_2_ is physically dissolved in PFCs and the release of oxygen from PFOB depends on the diffusion between oxygen concentration gradients [27]. This property also makes it difficult to retain high oxygen level in perfluorocarbon-based nanosystems for a long time in the natural state, which would impact the blood circulation time and the oxygen accumulation in tumor. Ensuring the O_2_ transport stability and rapidity of release was the key aspect for PFCs—based O_2_ carrier. It has been reported the near-infrared (NIR)-induced photothermal therapy (PTT) not only could kill the tumor [28–30], but also accelerate O_2_ release from PFCs—based O_2_ carrier to mitigate tumor hypoxia with remarkably synergistic effects [31, 32]. Therefore, sealing off the oxygen in a nanosystem and release it by NIR may be a strategy for delivering O_2_ to tumor.

**Scheme 1:**
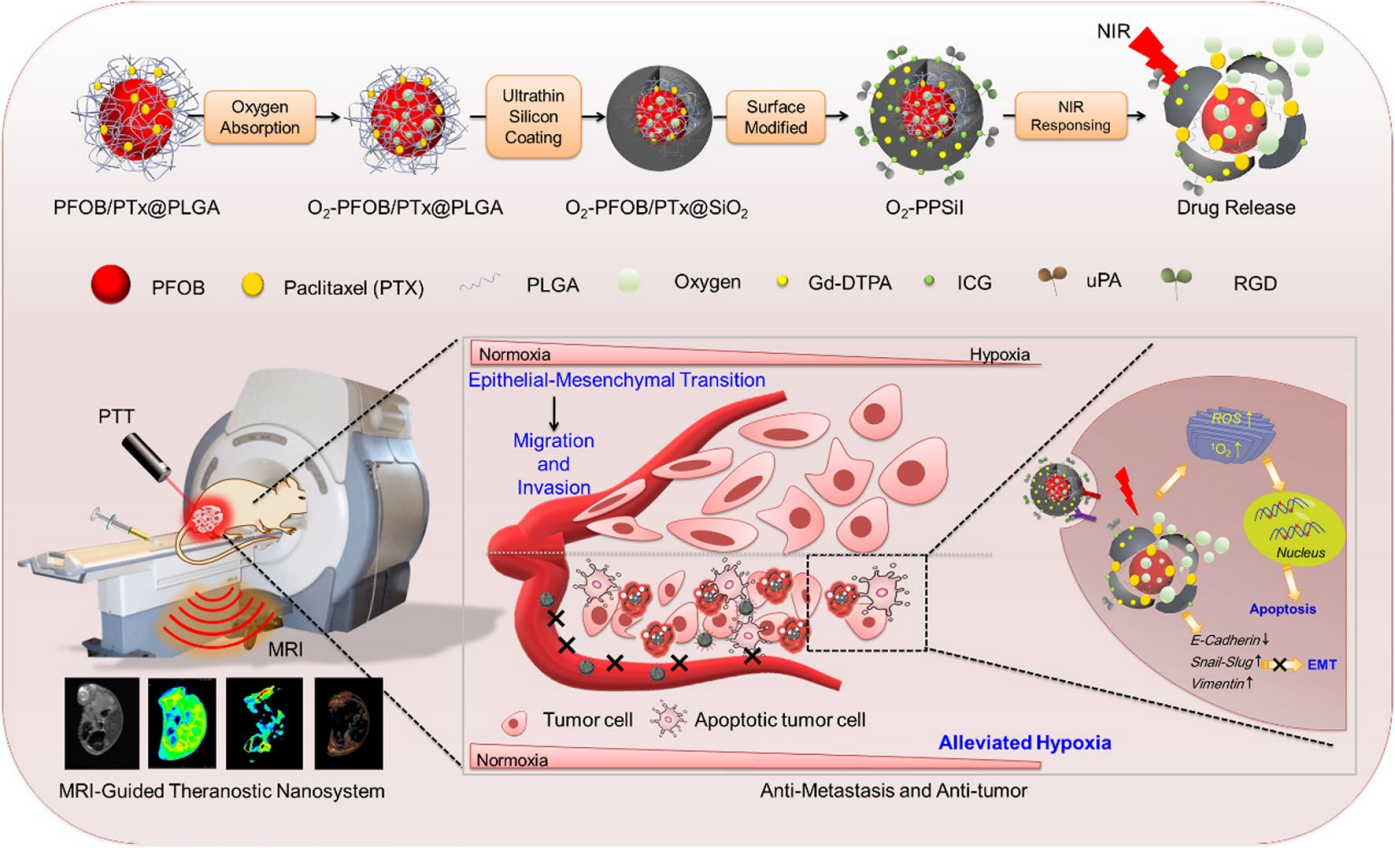
The rational design of NIR-responsive on-demand drug releasing nanomedicine system to relieve tumor hypoxia, enhance chemotherapy and inhibit tumor metastasis

Hence, a near-infrared responsive on-demand oxygen releasing nanoplatform (O_2_-PPSiI) was successfully established to delivery O_2_ to tumor and trigger its release under NIR irradiation. The poly(lactic-co-glycolic acid) (PLGA) was served as a core to load both oxygen carrier perfluorooctyl bromide (PFOB) and the chemotherapy drug PTx. Then, covering the ultrathin-walled silica shell on it was used to seal the O_2_ in this nanosystem. The Indocyanine green (ICG) served as a photothermal converter to trigger the rupture of silica shell and O_2_ release. Furthermore, in order to realize the precise and controllable on-demand drug releasing, the non-invasive radiation-free modality MRI and its T1 contrast agent paramagnetic gadolinium (Gd^3+^) complexes, which are all widely used for breast imaging in clinic, was applied to monitor the distribution of nanosystem in real-time. In addition, the Arginine–glycine–aspartic acid (RGD) and urokinase plasminogen activator (uPA), whose receptor integrin α_v_β_3_ and uPAR are overexpressed on human tumor cells [33, 34], were decorated on the surface of O_2_-PPSiI to transmit the drug into the tumor. The on-demand drug and oxygen release were achieved with an NIR-induced photothermal effect to realize precise treatment against TNBC via synergistic chemotherapy, PTT, and hypoxia mitigation; there were no toxic side effects. In addition, NIR-triggered O_2_-PPSiI could inhibit the natural procedure of epithelial-mesenchymal transition (EMT) in the TNBC and weaken its migration and invasion ability (Scheme 1). This study offers a smart “Trojan Horse” strategy guided by Gd-enhanced MRI and provides a valid approach for the reasonable design of an NIR-responsive on-demand drug-releasing nanosystem for precise theranostics in TNBC.

## Experimental section

### Materials

PLGA, PFOB, tetraethoxysilane (TEOS), and (3-aminopropyl) triethoxysilane (APTES) were bought from Aladdin Company (Shanghai, China). PTX was bought from Macklin Company (Shanghai, China), and ICG was bought from Sigma-Aldrich Company (St. Louis, MO USA). Commercial Gd-DTPA was bought from Shering AG (Berlin, Germany). Targeting ligands uPA and RGD were bought from Sigma-Aldrich Company (St. Louis, MO USA) and GL Biochem Ltd. (Shanghai, China), respectively. Fluorescent probes DHE and DPBF were bought from Sigma-Aldrich Company (St. Louis, MO USA). Antibodies against HIF-1α, Vimentin, Snail-Slug, and E-cadherin were bought from Abcam (Cambridge, UK). The water utilized in this study was distilled.

### Synthesis of O_2_-PPSiI

The synthesis of O_2_-PPSiI were described previously [17, 35]. Briefly, the 180 μL liquid PFOB and 20 mg PTX were both mixed into a 10 mL acetone of 70 mg PLGA to form a clear solution. The solution was then added into the cetyltrimethylammonium bromide (CTAB) solution (0.2 g/40 mL), emulsified by ultrasonic in ice-bath conditions for 10 min, and then completely volatilize acetone at the room temperature. The PFOB/PTX@PLGA nanoparticles (PP) were obtained.

Next, PP solution was dispersed into 45 mL distilled water and injected into 100 mL round-bottom flask under O_2_ atmosphere. Keeping the Stir at 200 rpm for 1 h made the nanoparticles fully absorb oxygen. 5 mL isopropanol was added by stirring for 30 min followed by the addition 0.5 mL TEOS and 0.01 mL APTES. After reacting for 12 h, the products were added into the dichloromethane solution with 50 mg DL-menthol by reaction for another 12 h at 35 ℃, and the O_2_-PFOB/PTX@SiO_2_ nanoparticles (O_2_-PPSiI) were collected by centrifugation. Afterwards, ICG, Gd-DTPA, and O_2_-PPSiI were mixed in an aqueous solution. After 12 h, uPA and RGD linked to the surface of silica shells with the catalysis of 1-ethyl-3-(3-dimethylaminopropyl) carbodiimide hydrochloride (EDC) and N-hydroxysuccinimide (NHS).

### Oxygen storage and release of O_2_-PPSiI

To examine the stability of the oxygen storage in O_2_-PPSiI, the nanoparticle was dispersed in the deoxygenated water and divided into seven samples for different times at room temperature. A portable oxygen meter (YSI-550A, YSI, USA) was employed to monitor the dissolved O_2_ concentrations in the solutions excited with an 808 nm NIR laser at a density of 1.5 W/cm^2^ for 6 min.

To examine the performance of oxygen release in O_2_-PPSiI. An ultrasound imaging device (Philips iU-Elite ultrasound system, USA) was used to visualize the process of oxygen release in O_2_-PPSiI, and the images of the sample or the tumor before and after irradiation were obtained at a gain of 69% or 79%, depth of 2.5 or 1.3 cm, and a mechanical index 0.6.

### Evaluation of cell migration and invasion

Cell migration was examined with a wound healing assay. Briefly, MDA-MB-231 cells (2 × 10^6^ cells/well) were seeded into the 6-well plates. After culturing for 24 h, each well was scratched in the middle of the cells. The medium was then taken away, and the cells were washed with PBS, and then the DMEM with 3% FBS was added. Next, the cells were exposed to different treatment groups with incubation for 24 h. After the addition of Hoechst 33,342 (1 μg/mL) for 30 min, the images of cell migration were obtained using a fluorescence microscope.

Cell invasion was examined with transwells assay. Briefly, a Boyden transwell chamber (Corning, USA) was added with dissolved ECM gel and incubated for 4–8 h at 37℃. MDA-MB-231 cells were added into the DMEM and seeded into the Boyden chamber (5 × 10^5^ cells/mL). A total of 500 mL DMEM with 10% FBS was put into the lower chamber. Next, different treatment agents at the same concentration were added into the chamber for 24 h at 37℃. Thereafter, the cells that invade through the upper chamber were fixed with methanol, stained with crystal violet, and observed with an inverted optical microscope (Olympus, Japan).

### Establishment of MDA-MB-231 orthotopic xenografts

All the animal study was performed with the approval of the Animal Experimentation Ethics Committee of Jinan University. Female BALB/c nude mice with 4–5 weeks of age were bought from Vitalriver Inc. (Beijing, China) and raised in a specific pathogen-free environment. The MDA-MB-231 cells at logarithmic growth phase were digested and collected as a suspension (2 × 10^5^ cells/40 μL) followed by injection into the fat pad of the third breast on the right to establish MDA-MB-231 orthotopic xenografts. After growing for about 28 days, the mice with the tumor reached approximately 300 mm^3^ and were included to perform the next experiments. All the mice were anesthetized with intraperitoneal injection of 2% pentobarbital (75 μL /200 g) sodium salt before the examinations.

### In vivo antitumor activity

To investigate the antitumor activity of O_2_-PPSiI, the included tumor-bearing mice were divided into 7 groups with the treatment of saline, laser, PTX, O_2_-PPSiI, PPSiI (the nanoparticles without O_2_) irradiated with NIR-laser (simplified as “PPSiI + Laser”), O_2_-PSiI (the nanoparticles without PTX) irradiated with NIR-laser (simplified as “O_2_-PSiI + Laser”) and O_2_-PPSiI irradiated with NIR-laser (simplified as “O_2_-PPSil + Laser”). All the agents were injected through the caudal vein at equivalent concentration of 10 mg/kg PTX, and the 808 nm NIR-laser was used at a density of 2 W/cm^2^ for 5 min. All the groups were treated twice a week with an interval of 3 days, and the NIR-laser irradiation was conducted at 8 h after the intravenous injection which was precisely identified according to the MR imaging of O_2_-PPSiI in vivo.

The structural and functional MRI including three-dimensional T2-weighted imaging (3D-CUBE T2WI), blood oxygenation level-dependent magnetic resonance imaging (BOLD-MRI) and intravoxel incoherent motion diffusion-weighted imaging (IVIM-DWI) sequences were performed to monitor the antitumor activity of O_2_-PPSil before and 7 days, 14 days and 21 days after the treatment with a 1.5 T Signa HDxt superconductor clinical Magnetic resonance system (GE Medical, Milwaukee, U.S.). The 3D-CUBE T2WI was scanned with TR/TE, 2000/83.1 ms; FOV 60 mm × 60 mm; matrix, 192 × 160; slice thickness/space, 1.5/0 mm; and the volume of each tumor was calculated through volume render program at the post-processing workstation (AW4.5, GE Healthcare). The BOLD-MRI was scanned with TR/TE, 235/3.9–104.7 ms; FOV 50 mm × 50 mm; matrix, 192 × 128; slice thickness/space, 2.0/0.2 mm; and the R2* was mapped through Functool R2star program at the post-processing workstation (AW4.5, GE Healthcare). The IVIM-DWI was scanned with TR/TE, 3000/101.7 ms; FOV 50 mm × 50 mm; matrix, 128 × 96; slice thickness/space, 2.0/0.2 mm; b values, 0, 25, 50, 75, 100, 150, 200, 400, 600, 800, 1000, 1200 and 1500 s/mm^2^; and then the diffusion-related parameter D and perfusion-related parameter f was mapped via the Functool MADC program at the post-processing workstation (AW4.5, GE Healthcare). Besides, the body weight of all the mice in each group was also recorded every two days. All the results were normalized to their percentage difference (△X (%) = (X^*i*^-X^*base*^)/X^*base*^ × 100%, which *i* represented the different time points, and X referred to the Volume, R2*, D, f and Body weight as mentioned above). After 21 days, all the mice in each group were sacrificed, and the tumor was separated and weighted, and organs and blood sample were also collected to conduct the hematoxylin and eosin (H&E) staining and biochemical analysis.

### Statistical analysis

All the data are presented with mean ± standard deviation (SD). Differences among groups were evaluated with one-way analysis of variance (ANOVA) and the least significant difference (LSD) t-test. Pearson correlation analyses between MRI-derived parameters and the expression of E-cadherin, vimentin, and Snail-Slug were performed. A difference of *P* < 0.05 (*) or *P* < 0.001 (**) was considered statistically significant. All statistical analyses were completed using the SPSS software package (Version 14.0, SPSS Inc., Chicago, IL, USA).

## Results and discussion

### Facile synthesis and characterization of O_2_-PPSiI nanosystem

**Fig. 1:**
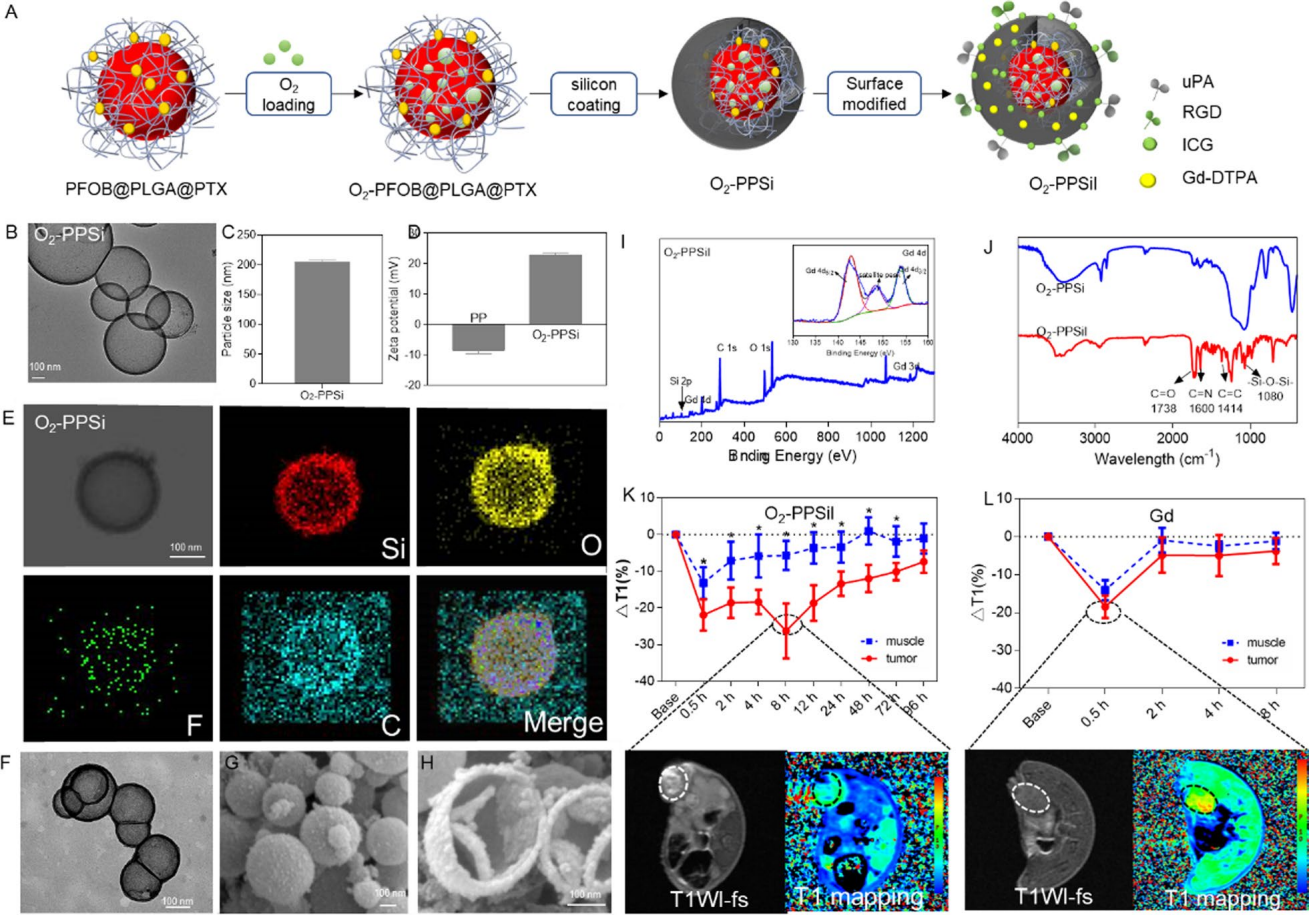
The Synthesis and structural characterization of O_2_-PPSiI. **A** Diagrams for synthetic process of O_2_-PPSiI. **B** TEM image of O_2_-PPSi. **C** The average sizes of O_2_-PPSi. **D** The zeta potential of PP and O_2_-PPSiI. **E** EDS element mapping images of O_2_-PIr@Si. **F** TEM image of O_2_-PPSiI. **G** The SEM image of O_2_-PPSiI. **H** The SEM of deliberately selected broken O_2_-PPSiI nanosystem. **I** The XPS of O_2_-PPSiI. The inserted data is the high resolution Gd 4d XPS spectra for O_2_-PPSiI. **J** The FTIR spectrum of O_2_-PPSi and O_2_-PPSiI. **K** and **L** The dynamic accumulation of O_2_-PPSiI and free Gd-DTPA in the tumor monitored by T1WI and T1 mapping-MRI, and significant difference between the muscle and tumor is indicated at *P* < 0.05 (*) level

The O_2_-PPSiI nanosystem was chemically synthesized by a two-stage self-assembly process, which was the PFOB core as the oxygen carrier, and then encapsulated into an ultrathin-walled silica shell (Fig. 1A). In the first stage, the PFOB core (i.e., the core without the silica coating) was synthesized via an emulsion–(solvent-evaporation) method [35], and PTx was mixed into PLGA to form the PFOB/PTX@PLGA (abbreviated as PP) nanocapsule. And then the PP nanocapsule was used to absorb O_2_ as a carrier. In the second stage, the ultrathin-walled silica shell was synthesized by hydrolysis and condensation of TEOS. The silica shell was heterogeneous in nature, and thus biocompatible DL­menthol was used to prevent oxygen release from the nanosystem. The loading efficacy of PTX in O_2_-PPSil was about 20%.

Transmission electronic microscopy (TEM) pictures presented the morphology of O_2_-PPSi which was a typical spherical shell/core structure (Fig. 1B) with an average diameter of 203 nm (Fig. 1C). Compared to PP (− 8.8 mV), the zeta potential of O_2_-PPSi elevated to + 22.9 mV (Fig. 1D), which is due to the formation of the aminated silica shell on the PP surface. The elemental mapping images of O_2_-PPSi nanoparticles showed the presence of Si and F elements (Fig. 1E), which further verified the loading of PFOB and coating of the silica shell. Benefiting from the rich amino and silicon hydroxyl groups on the surface, the tumor-targeting ligands uPA and RGD were covalently modified to the surface of O_2_-PPSi nanoparticles; photothermal agent ICG and MR contrast agent Gd-DTPA (Gd) were also added by electrostatic interaction. The morphology of the nanosystem (named O_2_-PPSiI) was also observed by TEM and scanning electron microscopy (SEM). Figure 1F and G show uniform O_2_-PPSiI with an average diameter of 203 nm, and the thickness of silicon shell with the decoration on its surface was about 8 nm (Fig. 1H).

The X-ray photoelectron spectroscopy (XPS) and Fourier transform infrared spectroscopy (FTIR) results further proved that the O_2_-PPSiI was successfully synthesized. The silicon and the gadolinium peaks were seen in the XPS survey spectra for the surfaces of O_2_-PPSiI, suggesting that the silica shell and Gd-DTPA were successfully coated and modified on the shell of O_2_-PPSiI (Fig. 1I). The high resolution Gd 4d XPS spectra for O_2_-PPSiI inserted in Fig. 1I revealed that the characteristic peaks centered at 142.8 eV and 153.7 eV could be attributed to Gd 4d_5/2_ and Gd 4d_3/2_, suggesting the existence of Gd (III), which derived from Gd-DTPA [36]. A satellite peak at 148.2 eV in the Gd 4d XPS spectra may derived from the coordinate bond of Gd (III) and DTPA. The XPS spectrum of C 1 s is shown in O_2_-PPSi (Additional file 1: Fig. S1A), and the peak components at 284.5 eV and 286.6 eV were appointed to the C–C and C–N groups, respectively. These were attributed to APTES on the surface of O_2_-PPSi. After ICG and Gd-DTPA grafting on the surface of O_2_-PPSi, two new peaks at an energy of 284.7 eV and 288.3 eV were seen and could be attached to the C = C and C = O group, respectively (Additional file 1: Fig. S1B). The chemical structure of O_2_-PPSiI was analyzed using FTIR. Versus the FTIR spectrum of O_2_-PPSi, the broad band at ≈1600 cm^−1^ resulted from the C = N band and the peaks at 1414 cm^−1^ that are represented by the vibrational stretching of the C = C groups (Fig. 1J), which are from the ICG. The spectrum of DTPA exhibited the characteristic peak of the asymmetric and symmetric carbonyl (C = O) stretch of anhydride at 1738 cm^−1^. Meanwhile, a characteristic UV absorption spectra peak of ICG emerged at 808 nm after the ICG was grafted on the surface of O_2_-PPSi (Additional file 1: Fig. S2), which matched the wavelength of the NIR laser applied for PTT. Versus the O_2_-PPSi, the color change of O_2_- PPSiI indicated that the ICG was coated on the surface (Additional file 1: Fig. S3).

We also evaluated the magnetic properties of O_2_-PPSiI using T1-weighted imaging (T1WI) and T1 mapping MRI, and the results showed that the T1WI signal of nanosystem was linear and concentration-dependent. The T1 relaxivity (r1) of O_2_-PPSiI to be 27.812 mM^−1^/s^−1^ (Additional file 1: Fig. S4), and it was dramatically superior than the free Gd-DTPA (4–5 mM^−1^/s^−1^), indicating a favorable T1WI contrast effect of O_2_-PPSiI. These results demonstrated that O2-PPSiI could enhance T1 positive contrast. Therefore, we further examined the accumulation of O_2_-PPSiI in vivo by MRI. The accumulation of O_2_-PPSiI in the tissue was quantitatively evaluated by the decrease of T1 value (longitudinal relaxation time, normalized as △T1 to the base). The injected O_2_-PPSil was selectively accumulated in the tumor and reached its maximum at 8 h (△T1 26.28%) after the systemic administration in vivo (Additional file 1: Fig. 1K). However, the accumulation of Gd-DTPA had no significant selectivity between the tumor and normal muscle tissue. It reached the maximum at 30 min (△T1 18.45%) but recovered to baseline after injection for 2 h (Fig. 1L). These results demonstrated that O_2_-PPSiI could enhance T1 positive contrast of Gd-DTPA and tumor targeting ability against TNBC in vivo.

### Photothermal ability of O_2_-PPSiI nanosystem

**Fig. 2:**
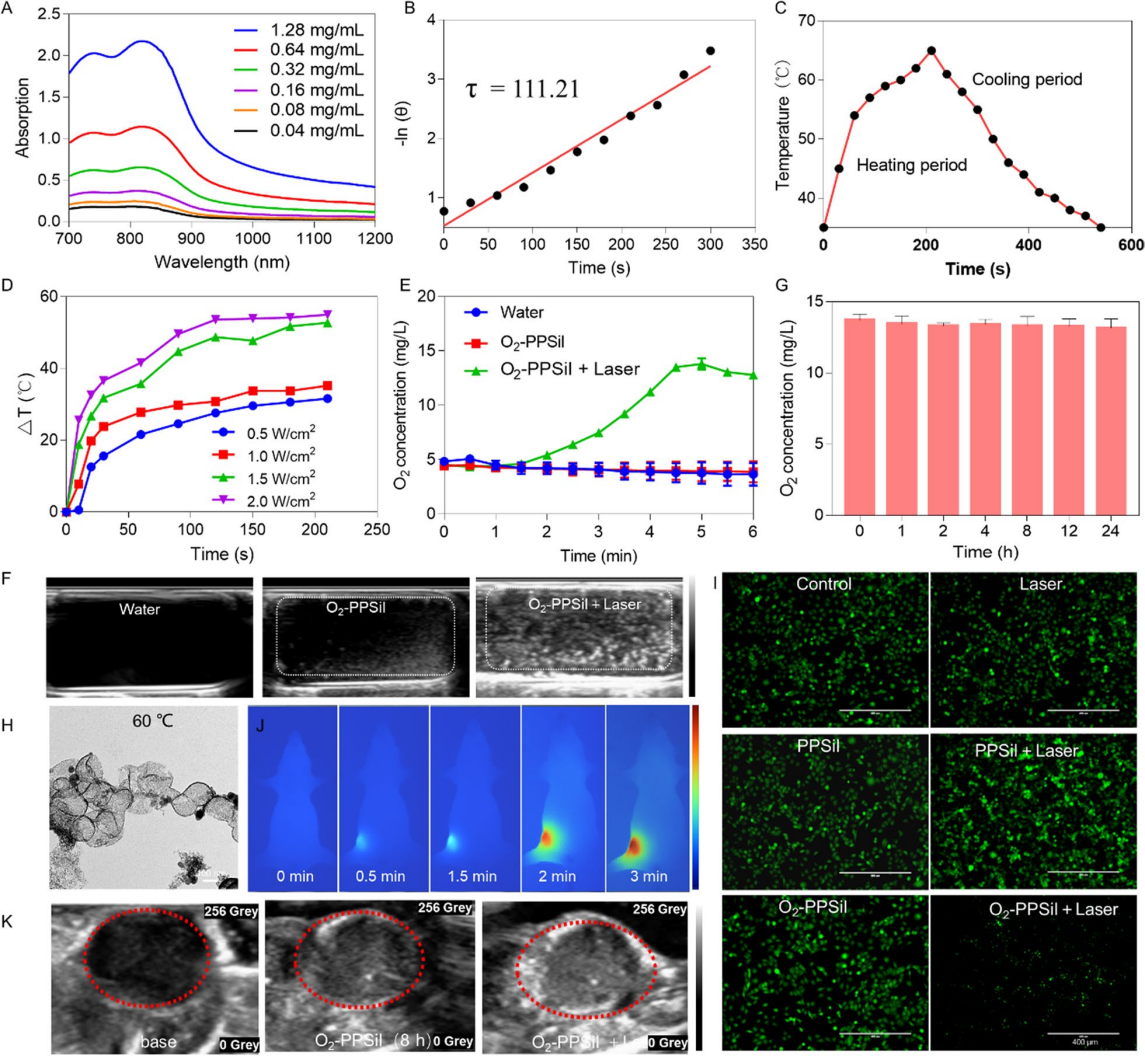
The oxygen release of O_2_-PPSiI triggered by NIR. **A** The UV–vis-NIR spectrum of O_2_-PPSiI at different concentrations. **B** Time constant for heat transfer from the O_2_-PPSiI was calculated to be 111.21 with the comparison of the linear time data deriveed from the cooling period to the negative natural logarithm of driving force temperature. **C** Photothermal effect of aqueous dispersion of O_2_-PPSiI under irradiation by NIR laser (808 nm, 2 W cm^−2^). **D** Photothermal-heating curves of O_2_-PPSiI in different powers. **E** The oxygen release from O_2_-PPSiI with NIR laser irradiation. **F** The O_2_ retention in O_2_-PPSiI nanosystem within 24 h. **G** Ultrasonography for the water and O_2_-PPSiI solution before and after the NIR laser irradiation in vitro. **H** TEM images of O_2_-PPSiI nanosystem at 60 ℃. **I** Fluorescence images (scale bar: 20 µm) of intracellular O_2_ variation after different treatment probed with RDPP **J** Photothermal pictures of the tumor region irradiated with 808 nm NIR laser (1.5 W/cm^2^) after 8 h of drug administration in the MDA-MB-231 tumor-bearing nude mice **K** Ultrasonography for the tumor at baseline, intravenously injected with O_2_-PPSiI before and after the NIR laser irradiation in vivo

The photothermal ability of O_2_-PPSiI was then examined in this study. The absorption of O_2_-PPSiI at 750 − 850 nm showed obvious concentration dependence (Fig. 2A). The extinction coefficient of O_2_-PPSiI at 808 nm for different concentrations was 82.16 Lg^−1^ cm^−1^ (Additional file 1: Fig. S5), indicating that the O_2_-PPSiI nanosystem has high photothermal-conversion performance due to ICG modification. The photothermal-conversion efficiency (η) of the O_2_-PPSiI nanosystem was determined as 45.45% based upon the data analysis in Fig. 2B and C. To estimate the photothermal-conversion performance of O_2_-PPSiI, it was stimulated by an 808 nm NIR laser with increased power densities (0.5, 1.0, 1.5, and 2 W cm^−2^) when the concentration was locked at 300 µg mL^−1^. As shown in Fig. 2D, the temperature elevation of O_2_-PPSiI increased by 35 °C (reached 61 °C) over 3 min of irradiation (1.0 W cm^−2^), and the thermal pictures of this process were documented with an IR thermal camera (Additional file 1: Fig. S6), demonstrating that O_2_-PPSiI had an excellent photothermal-conversion performance. The packaging of ICG in O_2_-PPSiI can contribute to the improvement of photostability versus free ICG, as proved with a reducible photothermal effect under repeated laser cycling for five cycles [37] (Additional file 1: Fig. S7).

### NIR-triggered O_2_ release and drug delivery

The system has NIR-induced photothermal effects from ICG and active oxygen release in PFOB [38]. We encapsulated the oxygen reservoir and antitumor agent into the nanocore and developed an NIR-responsive on-demand drug releasing nanomedicine system to overcome the drug leakage of conventional nano-based drug delivery system (NDDSs). We measured the O_2_ concentration change of O_2_-PPSiI in deoxygenated water under a nitrogen atmosphere and found that the O_2_ concentration increased significantly in the O_2_-PPSiI solution; many bubbles appeared with longer NIR irradiation times (Fig. 2E and Additional file 1: Fig. S8). Subsequently, ultrasound imaging was used to visualize the NIR-triggered O_2_ release and drug delivery in vitro. Figure 2F and Additional file 1: Fig. S9 show an apparent enhanced echo intensity in the O_2_-PPSiI solution after NIR irradiation. This was further confirmed via successful development of the NIR-responsive on-demand drug releasing nanomedicine system in this study. Meanwhile, the O_2_ concentration of the O_2_-PPSiI solution without NIR irradiation at room temperature showed no obvious change within 24 h (Fig. 2G), suggesting that the stability of O_2_-PPSiI was favorable. This proved that the O_2_-PPSiI has higher O_2_­storage ability than other O_2_­delivery nanosystems. The temperature elevation of O_2_-PPSiI with NIR laser irradiation was the key contributor of oxygen release from O_2_-PPSiI. Figure 2G and Additional file 1: Fig. S10 show that the morphology of O_2_-PPSiI swelled and then gradually collapsed at 60 ℃ and 70 ℃ when the reaction temperature reached 50 ℃. This indicates that the O_2_ release from O_2_-PPSiI was accompanied with the structural collapse of the SiO_2_ shell.

Subsequently, the O_2_ probe [Ru(dpp)_3_]Cl_2_ (RDPP) which is prone to luminescence quenching by oxygen was used to monitor cellular O_2_-evolving. As displayed in Fig. 2I, comparing with the control group and PPSiI (the nanosystem without O_2_) group, the MDA-MB-231 cells in O_2_-PPSiI + Laser group showed a weak green fluorescence signal because the NIR irradiation triggered O_2_-release from O_2_-PPSiI. The photothermal-conversion performance of O_2_-PPSiI and the O_2_ release from O_2_-PPSiI nanosytem in vivo were further investigated via an IR thermal camera and ultrasound imaging. The outward temperature of tumor treated with O_2_-PPSiI gradually increased from 35 to 60 °C along with the laser irradiation time came to 3 min (808 nm, 1 W cm^−2^; Fig. 2J**)**. This temperature increase could ablate the tumor. Moreover, the ultrasound images recorded the O_2_ release process under the laser irradiation (Fig. 2K and Additional file 1: Fig. S11). These results demonstrated that O_2_-PPSiI offered NIR-responsive on-demand drug-release in vitro and in vivo.

### Distribution of O_2_-PPSiI nanosystems in vivo

**Fig. 3:**
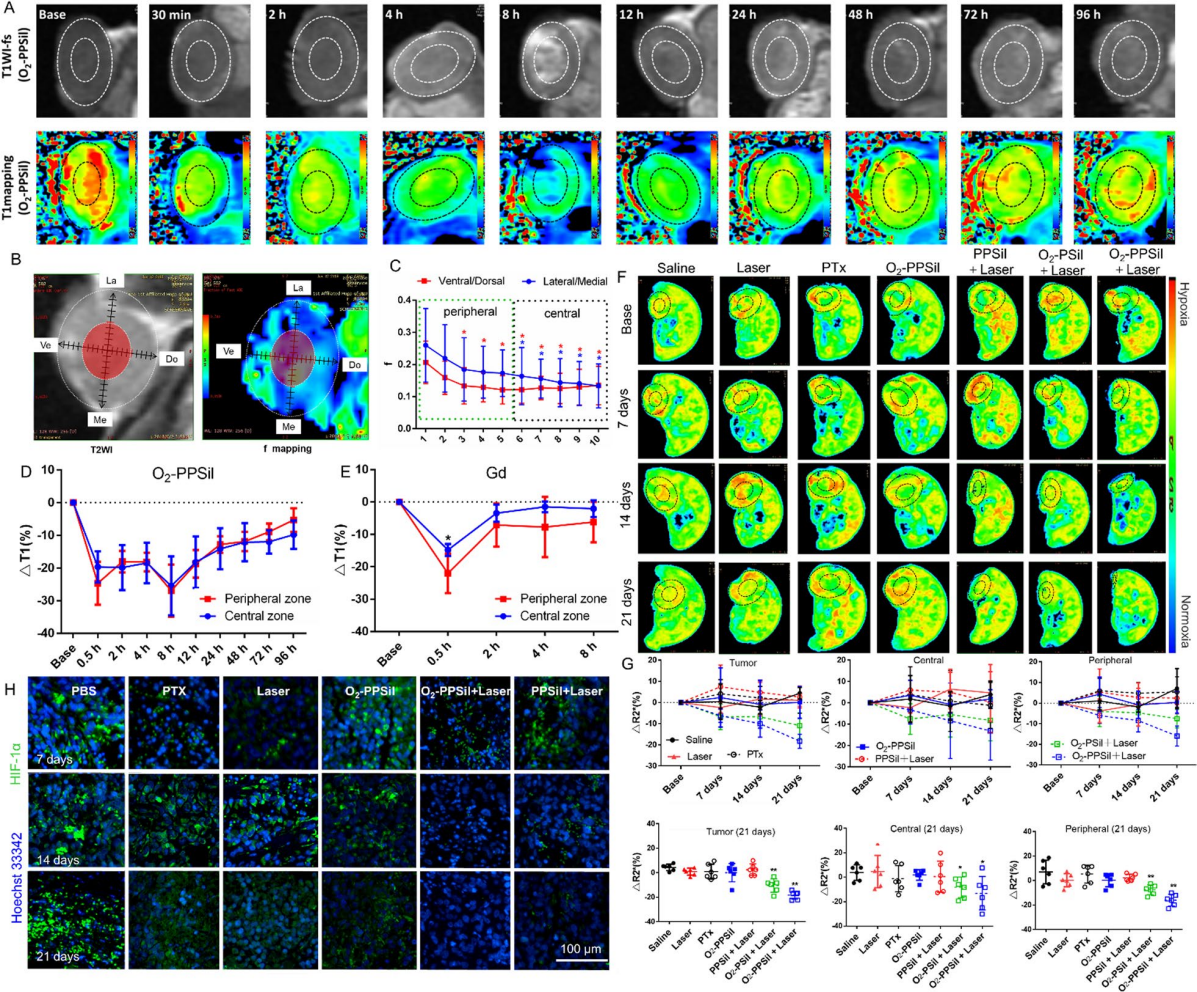
The distribution of O_2_-PPSiI in vivo. **A** The representative images of T1WI and T1 mapping-MRI for the dynamic accumulation of O_2_-PPSil in tumor. **B**, **C** The confirmation of tumor central and peripheral zones based on IVIM-DWI derived f mapping, and significant difference between the outermost segment (“1”) and inner segments (“2–10”) is indicated at *P* < 0.05 (*) level. (**D**, **E**) The dynamic accumulation of O_2_-PPSil and free Gd-DTPA in the tumor central and peripheral zones monitored, and significant difference between the peripheral and central tumor is indicated at *P* < 0.05 (*) level. (**F**, **G**) The representative images for R2* mapping and the relative changes of R2* value (△R2*) of the whole tumor, central tumor and peripheral tumor in each groups before and after the treatments, and significant difference between the Saline and treatment groups is indicated at *P* < 0.05 (*) or *P* < 0.001 (**) level. **H** Representative immunofluorescence pictures of tumor slices stained with the Anti-HIF-α (green)

The targeting efficacy of the nanoparticle to by RGD and uPA to TNBC was investigated. We firstly compared the intracellular uptake of O_2_-PPSiI between MDA-MB-231 cells and normal breast cells (Hs 578Bst), and the O_2_-PPSiI exhibited much higher cellular uptake in MDA-MB-231 cells, but obvious lower in normal breast cells (Additional file 1: Fig. S12A and B). And then, the targeted molecule competition assay further proved that the highly selective intracellular uptake of O_2_-PPSiI in MDA-MB-231 cells was mediated by the RGD and uPA (Additional file 1: Fig. S12C). In addition, the targeting efficacy in vivo of the nanoparticle to by RGD and uPA to triple negative breast cancer was detected by using nanosystem with or without different targeting molecule the bio fluorescence imaging. As shown in Additional file 1: Fig. S12D, the double targeted-nanosystem (O_2_-PPSiI with uPA and RGD) has a higher accumulation in tumor than the O_2_-PPSiI nanosystem with uPA or RGD. These results proved the design of double targeting molecule could result in the high targeting efficacy of O_2_-PPSiI to TNBC. Subsequently, MRI was used to monitor the distribution of the O_2_-PPSiI nanosystem in vivo at various time points. Figure 3A shows that intravenously injected O_2_-PPSiI was selectively accumulated in the tumor region and came to its maximum at 8 h. Therefore, dual modal imaging can effectively and precisely monitor the distribution of the O_2_-PPSiI nanosystem in vivo and confirm the best time point of NIR irradiation.

Transporting the drugs to the deep tumor tissue is limited owing to abnormal angiogenesis and irregular tumor blood flow. Currently, functional nanosystems have provided new strategies to enhance tumor penetration and achieve effective and successful antitumor therapy [39]. Here, intravoxel incoherent motion diffusion-weighted imaging (IVIM-DWI) was used to evaluate the in vivo tumor vascularity. As a non-contrast-based functional MRI sequence, IVIM-DWI is an attractive approach to assess not only the tumor cellularity but also the micro-perfusion with no need to use exogenous contrast agents. This allows it to be repeatedly used as a method of quantitatively monitoring the therapeutic response in vivo [40]. In this work, we applied IVIM-DWI to evaluate the microperfusion of the baseline tumors, and then drew two orthogonal lines in the Dorsal/Ventral (Do/Ve) and Medial/Lateral (Me/La) directions crossing the tumor center. This divided each radius into 10 segments with equivalent lengths (each segment was then appointed with a figure of 1–10, i.e., the outermost segment was appointed with 1 and the innermost segment of 10 at a radial position). We then confirmed the heterogeneous vascularity of TNBC and found that the intratumor blood flow at the normalized radius ≥ 6 in both the Ve/Do and La/Me direction was significantly decreased versus the outermost segment (Fig. 3B and C), which was also observed by Gaustad [41]. Based on these results, we further divided the entire tumor into peripheral (the outermost segment 1 to segment 5) and central (segment 6 to the innermost segment 10) zones. The injected O_2_-PPSiI infiltrated deep into the central region with no significant difference in △T1 versus the peripheral zone (Fig. 3D). The Gd-DTPA mainly accumulated in the peripheral region and presented a lower △T1 than the central zone (Fig. 3E). These results confirmed a favorable intratumor penetration of O_2_-PPSiI, and the potential mechanism may be associated with the cell-to-cell transport through the active targeting of uPAR in the tumor and stromal cells [42].

We further investigated the biodistribution of injected O_2_-PPSiI in the tumor-bearing nude mice. The T1 MRI data presented that, except for the intratumor accumulation, the injected O_2_-PPSiI was mainly collected by the kidney within 24 h (Additional file 1: Fig. S13A). It had a fluctuating yet increasing trend in △T1 after 24 h (Additional file 1: Fig. S13B), which may reflect the urinary excretion of O_2_-PPSiI. In addition, the injected O_2_-PPSiI was also taken up by the liver and spleen that showed a fluctuating change in △T1 (Additional file 1: Fig. S14C–D). Moreover, the fluorescence images showed most of the injected O2-PPSil accumulated in the intestinal system within 8 h, and then vanished gradually after 12 h excepted for the retention in the tumor region. These results indicated a urinary and intestinal excretion of the O_2_-PPSiI, further confirming its favorable biodegradability in tumor theranostics.

### Antagonizing tumor hypoxia statues by NIR-triggered oxygen release in vivo

Hypoxia offers multiple problems in the treatment of cancers [43]. Antagonizing tumor hypoxia statues is important for tumor therapy [44, 45]. Therefore, the efficacy of relieving tumor hypoxia for O_2_-PPSiI nanosystems was further investigated with MDA-MB-M231 tumor­bearing nude mice. First, based upon the paramagnetic effect of deoxyhemoglobin, we used blood oxygenation level-dependent magnetic resonance imaging (BOLD-MRI) to monitor the real­time improvements in tumor hypoxia as well as the efficacy of NIR-triggered oxygen-shuttle nanomedicine in promoting tumor oxygenation. Improvements in tumor hypoxia were evaluated quantitatively using the decreased R2* values of BOLD-MRI [46]. Figure 3F shows that the tumors treated with NIR-triggered O_2_-PPSiI and O_2_-PSiI (without PTX, 808 nm, 1 W cm^−2^) demonstrated a reduction in R2* (normalized as △R2* to the base) versus the baseline in both the central and peripheral zones. These results proved a satisfactory tumor oxygenation level in the local tissue. Versus the saline group, the tumors treated with a single NIR laser, PTX, and O_2_-PPSiI showed no significant difference in △R2*, while the NIR-triggered O_2_-PPSiI and O_2_-PSiI treatment groups had a significant decrease in △R2* at the endpoints of the treatment in both the central and peripheral zone (Fig. 3G). The tumor treated with NIR-triggered PPSiI was considered as a negative control to verify the contribution of O_2_ release for the tumor hypoxia relieving effect. Versus the NIR-triggered PPSil group, the tumor treated with NIR-triggered O_2_-PPSil showed a significantly decreased △R2* in both the central and peripheral zones, which further proved the improvements in tumor hypoxia statues by NIR-triggered oxygen release [47]. Furthermore, we performed HIF-1α immunofluorescence staining to confirm the efficacy of NIR-triggered O_2_-PPSiI in promoting tumor oxygenation (Fig. 3H). Consistent with the results of BOLD-MRI, the tumor treated with NIR-triggered O_2_-PPSiI showed a decreased expression of HIF-1α. But then, tumors treated with single NIR, PTX, O_2_-PPSiI, and NIR-triggered PPSiI exhibited high expression of HIF-1α with strong green immunofluorescence in different levels. The BOLD-MRI and immunofluorescence staining data suggested that NIR-triggered O_2_-PPSiI could mitigate the hypoxia microenvironment of TNBC in vivo, especially to the central tumor that presented as an avascular region with poor blood flow.

### Synergistic therapeutic efficacy of NIR-triggered O_2_-PPSiI

The in vitro cytotoxicity of the O_2_-PPSiI nanosystem was investigated with MDA-MB-231 cells by MTT [48]. Additional file 1: Fig. S14 shows that the viability of the cell treated with NIR irradiated O_2_-PPSiI reduced to 17.8%. Meanwhile, the MDA-MB-231 cells treated with PTX and NIR irradiated PPSiI and O_2_-PSiI exhibited higher cell viabilities of 55.9%, 35.1%, and 48.2%, respectively. The excellent cancer cell killing efficiency for NIR irradiated O_2_-PPSiI nanosystem in vitro could be attributed to the hyperthermia of ICG and the chemotherapeutic effect of PTX releasing from the O_2_-PPSiI nanosystem.

**Fig. 4:**
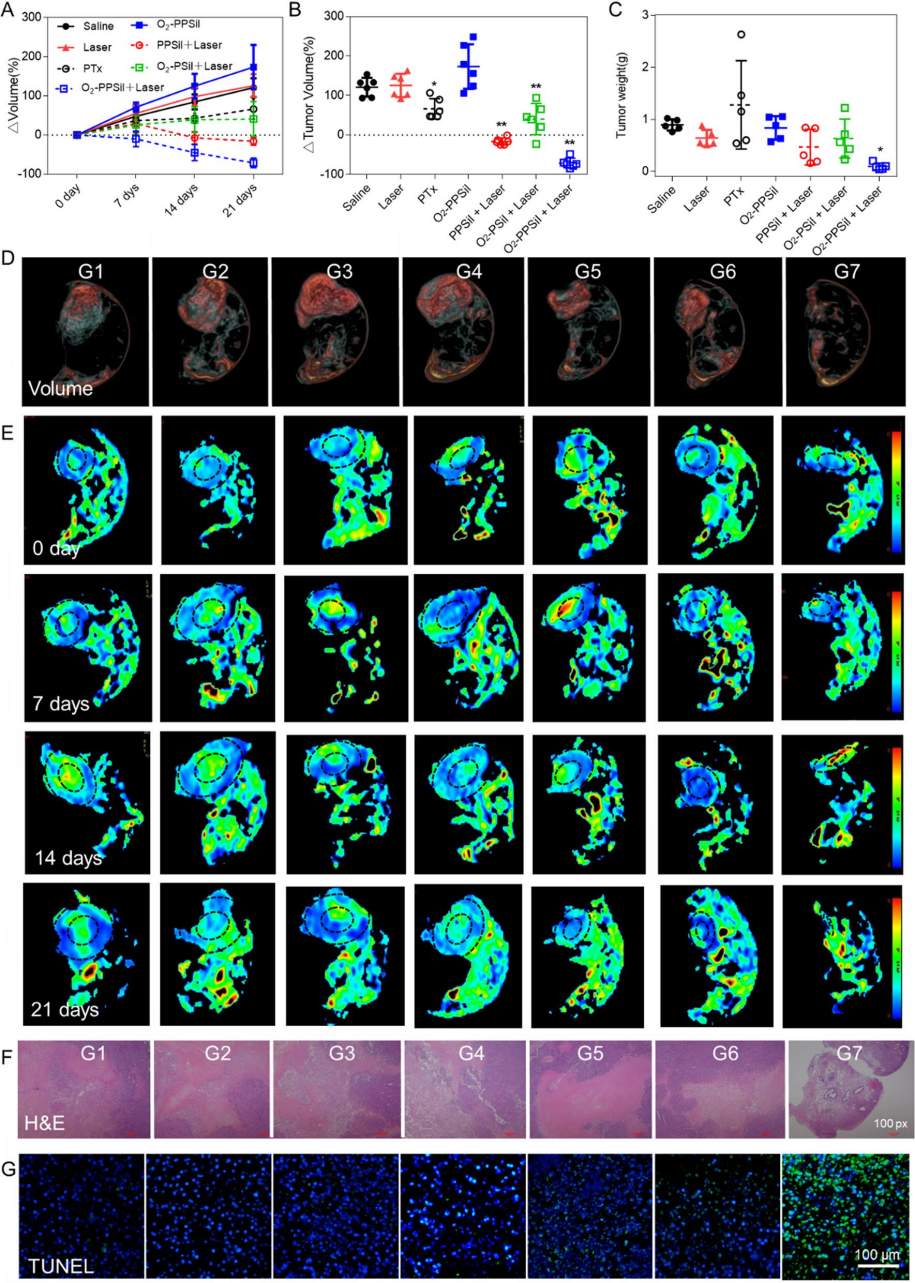
In vivo antitumor therapeutic efficacy for NIR-triggered O_2_-PPSiI. **A–C** The relative changes of volume (△Volume) in each groups before and after the treatments, together with the △Volume and weight of tumors of each groups at the 21 days after the treatment. Significant difference between the Saline and treatment groups is indicated at *P* < 0.05 (^*^) or *P* < 0.001 (^**^) level. **D** The representative images for tumor volume derived from 3D-CUBE T2WI in each group at the 21 days after the treatment. **E** IVIM-DWI derived D mapping of tumor in each group before and after the treatment. **F**, **G** The corresponding HE and TUNEL staining images at the 21 days after the treatment. (G1:Saline; G2:Laser; G3:PTX; G4: O_2_-PPSiI; G5:PPSiI + Laser; G6:O_2_-PSiI + Laser; G7: O_2_-PPSiI + Laser)

To further investigate the cancer cell killing mechanism of O_2_-PPSiI in vitro, we measured the ROS and ^1^O_2_ generated by O_2_-PPSiI [49]. Versus PTX and NIR-triggered PPSiI, O_2_-PPSiI under NIR laser irradiation showed more high-efficiency ROS and ^1^O_2_ generation in solution and MDA-MB-231 cells (Additional file 1: Fig. S15), indicating an important role of O_2_ release in the production of ^1^O_2_ and ROS [50, 51]. Subsequently, the in vivo synergistic antitumor effect of the NIR-triggered O_2_-PPSiI nanosystem was further investigated with MDA-MB-231-bearing nude mice. Based on the precise monitoring for the distribution of O_2_-PPSiI with T1 mapping MRI in vivo (Fig. 3A), the NIR irradiation was triggered at 8 h after the injection of PPSiI, O_2_-PSiI, or O_2_-PPSiI. The relative changes of tumor volume and weight are revealed in Fig. 4A–C. Over 21 days of treatment, the relative tumor volume (normalized as △Volume to the base) of mice treated with only saline, O_2_-PPSiI, and NIR irradiation groups increased significantly (Fig. 4A). The mice treated with PTx and NIR-triggered O_2_-PSiI (without PTX) presented a slightly increased tumor volume compared to day 0, demonstrating the moderate tumor inhibition of the single chemotherapy and phototherapy against TNBC. However, the tumor volume of mice treated with NIR-triggered PPSiI showed no significant difference at 14 days and 21 days compared to day 0, while the NIR-triggered O_2_-PPSiI group showed significantly decreased tumor volumes (Fig. 4B), and the volume as well as the tumor weight at the endpoint of both the groups were significantly lower than the PTX and NIR-triggered O_2_-PSiI groups (Fig. 4C), demonstrating an obvious inhibition and regression effect of the synergistic chemo-phototherapy. After treatment for 21 days, the picture of separated tumors in each group also presented similar results as the tumor volume and weight revealed above (Additional file 1: Fig. S16). Moreover, the tumor volume and weight of the mice treated with NIR-triggered O_2_-PPSiI were significantly lower than those treated with NIR-triggered PPSiI after 21 days coincided with the results of 3D-CUBE T2WI at 21 days (Fig. 4D). The excellent antitumor efficiency in vivo for the NIR-triggered O_2_-PPSiI nanosystem could be ascribed to an enhanced synergistic chemo-phototherapy efficiency induced by O_2_ release triggered by NIR laser irradiation.

To further investigate the antitumor effect of the NIR-triggered O_2_-PPSiI in vivo, the parameters of IVIM-DWI (D and f value) were applied to track the tumor cellularity and perfusion during the therapy [52]. Figure 4E and Additional file 1: Fig. S17 showed the △D value (normalized as △D to the base) in both the central and peripheral regions of the NIR-triggered O_2_-PPSiI group increased significantly compared the PTX, NIR-triggered O_2_-PSiI, and PPSiI groups, which demonstrated a reduced cellular density and activity of the tumor in vivo [53]. The results suggest that the enhanced synergistic chemo-phototherapy efficacy induced by tumor hypoxia improvement under NIR laser irradiation could effectively inhibit the tumor cell proliferation especially to the avascular central tumor. Moreover, tumor progression was suppressed by the synergistic chemo-phototherapy which was confirmed with microscopic analysis of tumor tissues stained by hematoxylin and eosin (H&E). Figure 4F shows that O_2_-PPSiI under NIR laser irradiation could effectively decrease the cell viability in tumor regions which offering an additional support for the antitumor efficacy of the nanosystem in vivo. Terminal deoxynucleotidyl transferase­mediated dUTP nick end­labeling (TUNEL staining assay) was also completed to further determine the cell proliferation and apoptosis in tumor tissue at treatment of 21 days. As displayed in Fig. 4G, the green and blue fluorescence of the NIR-triggered O_2_-PPSiI (G7) group had excellent overlap confirming DNA fragmentation and large apoptosis areas of tumor tissue.

### The suppression of tumor metastasis

**Fig. 5:**
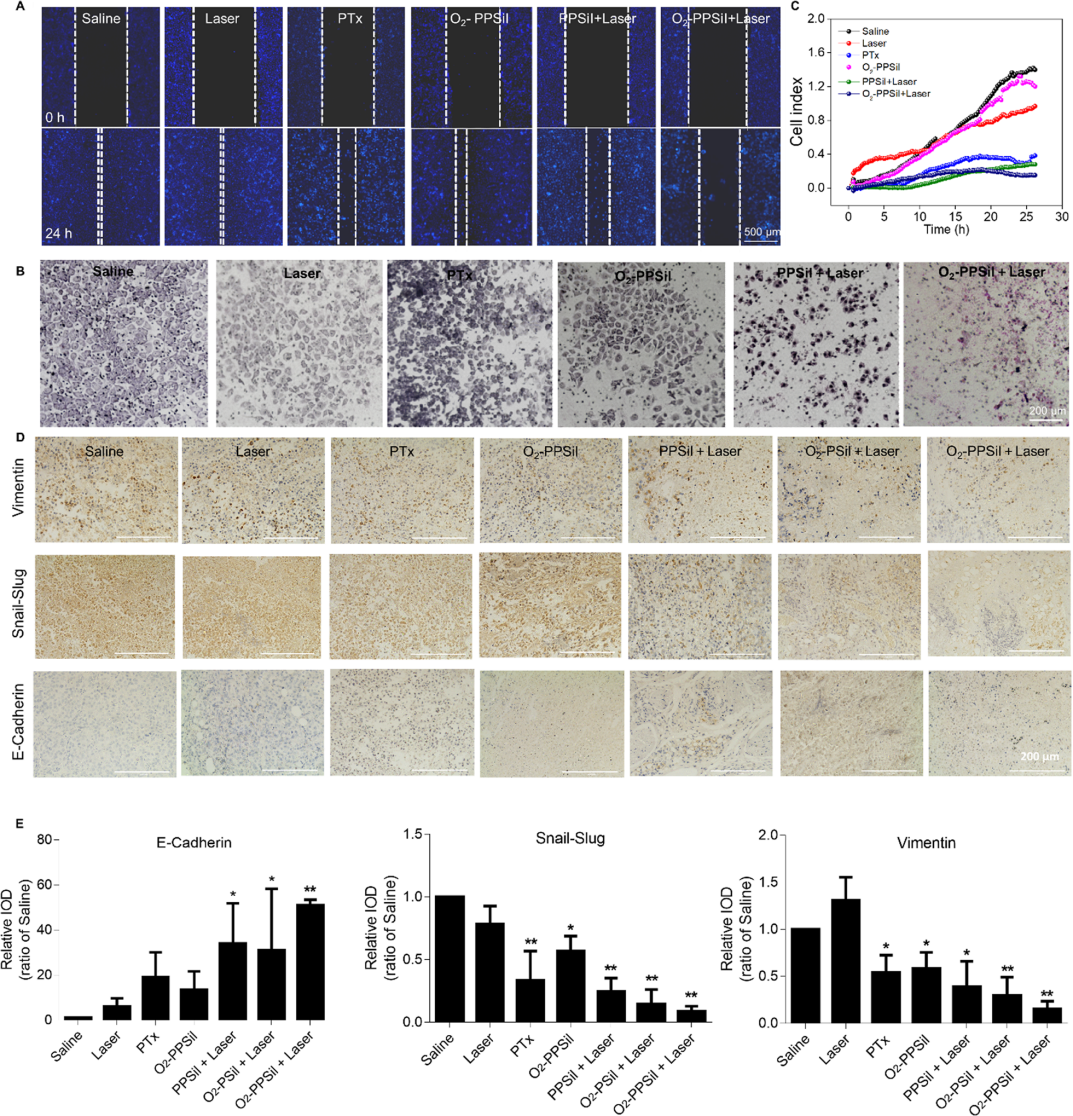
Anti-migration and anti-invasion effects of NIR-triggered O_2_-PPSiI. **A** Wound healing assay of each group on MDA-MB-231 cells. **B** Transwell assay of each groups on MDA-MB-231 cells. **C** Real-time monitoring the cell invasion of each group on MDA-MB-231 cells. **D** The immunohistochemistry for the epithelial-specific marker E-cadherin and the mesenchymal-specific markers vimentin and Snail-Slug in each group at 21 days after treatment. **E** The relative integrated optical density (IOD) of E-cadherin, vimentin and Snail-Slug in each group at 21 days after treatment, and significant difference between the Saline and treatment groups is indicated at *P* < 0.05 (^*^) or *P* < 0.001 (^**^) level

The metastasis of the tumor is a major concern in the clinic. It has been reported that the hypoxic microenvironment in the tumor could influence the progression of metastasis [54]. The combination of O_2_-PPSiI and the NIR laser could release the O_2_ to relieve tumor hypoxia due to the rupture of the silica shell, which might antagonize hypoxia-induced tumor metastasis. Therefore, we first used the wound-healing migration assay together with the transwell assay to evaluate the suppression effect of the tumor metastasis in vitro of O_2_-PPSiI under NIR laser irradiation. Figure 5A compares the PTX group and the PPSiI + NIR laser group. The O_2_-PPSiI under NIR laser irradiation could effectively suppress the migration of MDA-MB-231 cells at the same concentrations. Meanwhile, the invasive ability of MDA-MB-231 cells treated by different group was also detected and monitored in real-time. Versus the control (refer to the Saline and Laser group), the invasive ability of MDA-MB-231 cells treated by PTX, PPSiI + NIR laser, or O_2_-PPSiI + NIR laser was obviously decreased (Fig. 5B and C). Certainly, the combination of O_2_-PPSiI and NIR laser showed more effective suppression than PTX, PPSiI, and NIR laser. These results proved a synergistic effect of NIR triggered O_2_-PPSiI nanosystem on suppressing migration and invasion of MDA-MB-231 cells, and suggested a positive role of the NIR-triggered oxygen release and tumor hypoxia improvement.

In the tumor hypoxic microenvironment, signaling pathways that facilitate cell survival and metastasis are activated to give the tumor cell the ability to migrate and invade via the epithelial-mesenchymal transition (EMT) [55, 56]. EMT is a biological process that promotes the transformation of immotile epithelial cells to motile mesenchymal cells, involving a reduction of epithelial markers and the increase of mesenchymal markers in the tumor [57]. To further explore the effect of NIR-triggered O_2_-PPSiI on suppressing the tumor metastasis in vivo, the epithelial-specific marker E-cadherin together with the mesenchymal-specific markers vimentin and Snail-Slug were chosen to perform immunohistochemistry microscopy [58]. Figure 5D and E show that the expression of epithelial-specific marker E-cadherin in the tumor treated with NIR-irradiated O_2_-PPSiI was much higher than that of the single PTX group, O_2_-PSiI + Laser group, or PPSiI + Laser group. In addition, the expression of mesenchymal-specific markers Snail-Slug and vimentin in the tumor treated with NIR-irradiated O_2_-PPSiI was much lower than that of the single PTX group, O_2_-PSiI + Laser group, or PPSiI + Laser group. These results demonstrated that the NIR-irradiated O_2_-PPSiI could inhibit the process of EMT in the tumor and decrease its migration and invasion due to the tumor hypoxia improvement induced by the oxygen release under NIR laser irradiation and synergistic chemo-phototherapy. These effects were further verified by the results of correlation analysis presented in Additional file 1: Fig. S18. In addition, the uPA/RGD dual-targeting molecules of O_2_-PPSiI may play a positive role in this process as indicated by significantly downregulating the expression of mesenchymal-specific markers vimentin and Snail-Slug in the O_2_-PPSiI group compared to the Saline [58, 59].

**Fig. 6:**
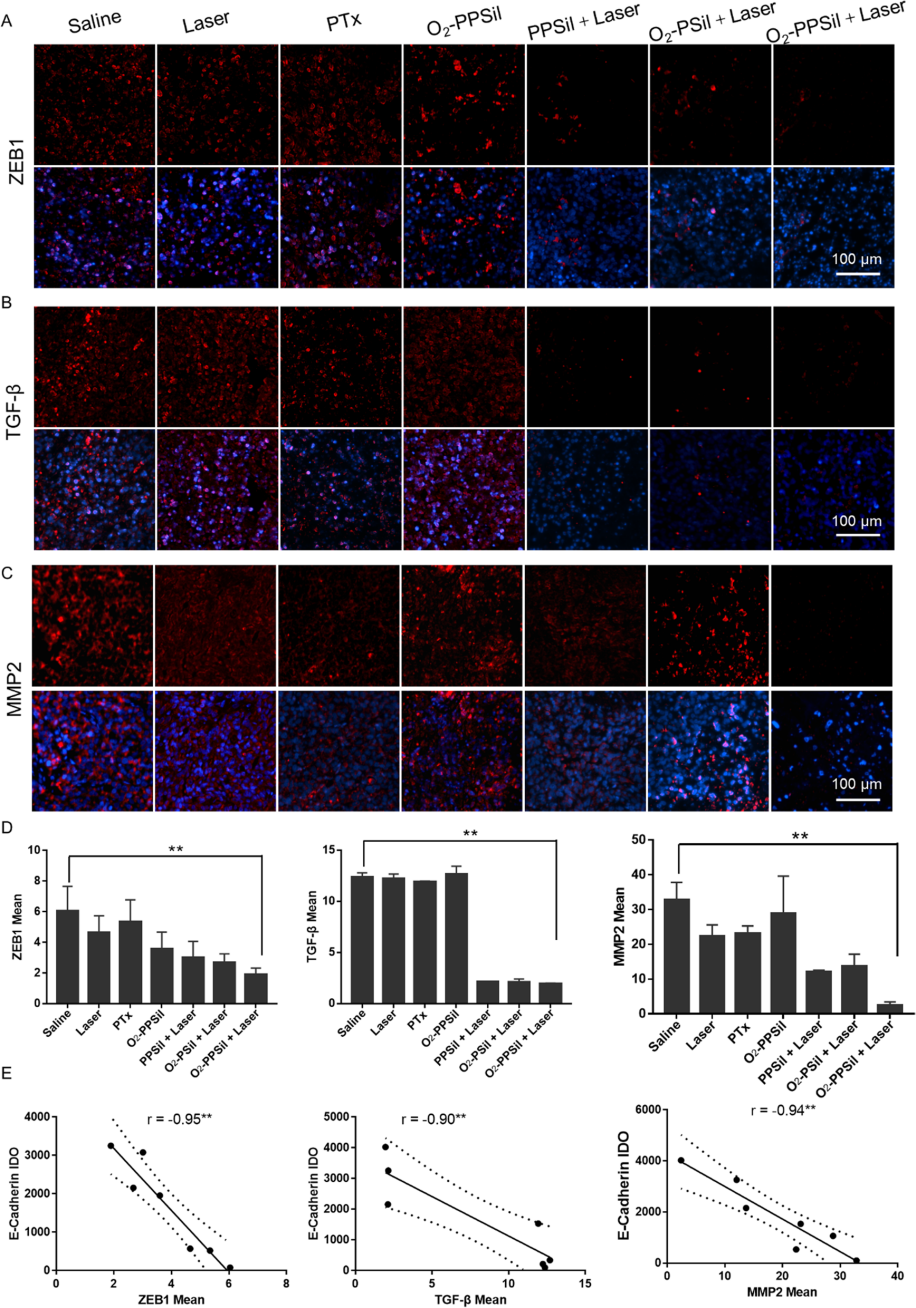
Action mechanism of anti-migration and anti-invasion effects caused by O_2_-PPSiI. **A**–**C** Immunohistochemical analysis of ZEB1, TGF-β and MMP2 expression in tumor. **D** The Fluorescence (red) quantitative analysis of ZEB1, TGF-β and MMP2. **E** Pearson correlation analysis between the expression of E-cadherin and ZEB1, TGF-β and MMP2, statistically significant correlation coefficient (r) is indicated at *P* < 0.05 (^*^) or *P* < 0.001 (^**^) level

Furthermore, the partial activation of EMT program was considered a major driver of tumor progression from initiation to metastasis. Particularly, zinc finger E-box binding homeobox 1 (ZEB1) and transforming growth factor β (TGF-β) play essential roles in the proliferation, migration, and invasion of tumor cells. We next examined whether O_2_-PPSiI could inhibit ZEB1 and TGF-β levels in orthotopic MDA-MB-231 tumor-bearing mice. Immunofluorescence for the ZEB1 verified that O_2_-PPSiI combined with NIR laser could efficiently reduce the expression of ZEB1 withintumor in comparison with the saline group suggesting that this combined strategy suppressed the activation of cell motility and stemness (Fig. 6A and D). Versus the mice in PPSiI + Laser group, the O_2_-PPSiI combined with NIR laser distinctly inhibited the expression of ZEB1, which confirmed that relieving tumor hypoxia could effectively abrogate the hypoxia-induced EMT. Meanwhile, the EMT was also reduced by TGF-β downregulation due to the synergistic effects of NIR-irradiated O_2_-PPSiI (Fig. 6B and D).

Matrix metalloproteinase-2 (MMP2) is a member of the zinc-binding endopeptidase family and plays an essential role in the invasion and metastasis of cancer cells. Upregulation of MMP2 expression can also promote tumor metastasis [60]. Therefore, we further detected the expression of MMP2, proving that O_2_-PPSiI combined with NIR laser could inhibit tumor metastasis. Figure 6C and D show that O_2_-PPSiI under NIR laser irradiation could effectively downregulate the MMP2 expression. The negative correlation analysis of E-cadherin, vimentin, Snail-Slug and ZEB1, TGF-β, and MMP2 demonstrated that the O_2_-PPSiI + NIR laser group could effectively influence the EMT (Fig. 6E and Additional file 1: Fig. S19). Therefore, these results indicated that O_2_­delivering strategies could alleviate hypoxia of the tumor tissue and abrogate the hypoxia-induced EMT to inhibit tumor metastasis.

### Biosafety evaluation of NIR-triggered O_2_-PPSil in vivo

**Fig. 7:**
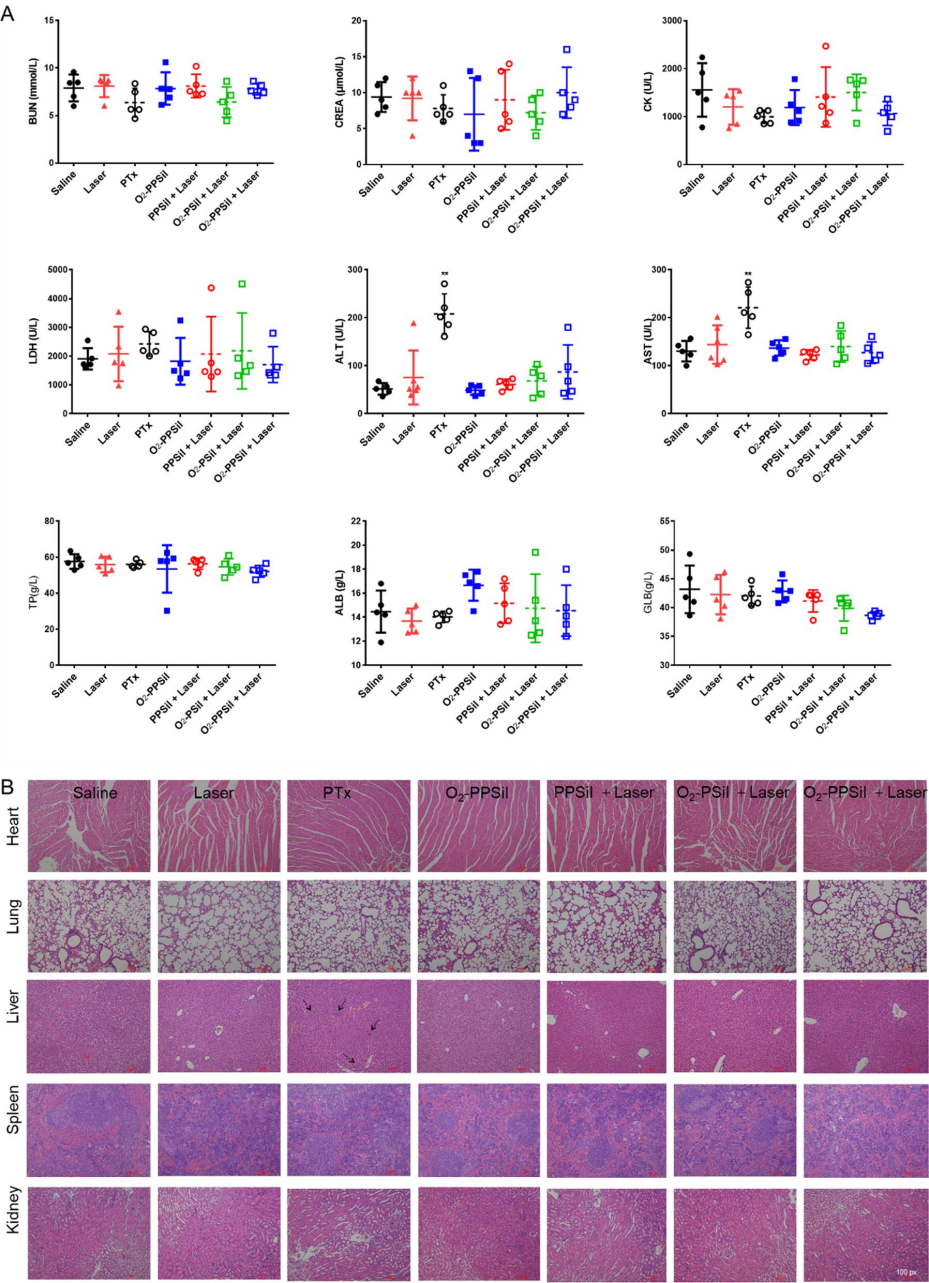
**A** The results of blood biochemical analysis in each group at 21 days after treatment, and significant difference between the Saline and treatment groups is indicated at *P* < 0.05 (*) or *P* < 0.001 (**) level. **B** The H&E staining of heart, lung, liver, spleen and kidney in each group at 21 days after treatment. The black arrows pointed to the region of patchy lymphocyte infiltration and hepatocyte swelling

The in vivo biosafety for O_2_-PPSiI was systematically inspected via body weight monitoring, blood biochemical analysis, and H&E staining [61]. Additional file 1: Fig. S20 shows that the body weight (normalized as △Body weight to the base) of the mice in NIR-triggered O_2_-PPSiI group increased during treatment for 21 days. This was significantly higher than the baseline and the saline group at 21 days. The body weight of PTX-treated mice declined significantly compared to the baseline over 9 days after injection. The blood biochemical analysis of mice treated with PTX also indicated an impaired metabolic function of the liver with significantly elevated Alanine transaminase (ALT) and Aspartate aminotransferase (AST) in the blood (Fig. 7A). This was further verified by the presence of patchy lymphocyte infiltration and hepatocyte swelling in liver H&E staining (Fig. 7B); no obvious hematological and histological abnormalities were observed in the other groups (Fig. 7A and B). These results indicated the hepatotoxicity of single PTX treatment and proved the safety of O_2_-PPSiI for clinical translation.

## Conclusion

Herein, the near-infrared responsive on-demand oxygen releasing nanoplatform O_2_-PPSiI was chemically synthesized in this study by a two-stage self-assembly process, which could deliver oxygen and release it under NIR irradiation to relieve hypoxia. This improved the therapeutic effect of chemotherapy and suppressed tumor metastasis. This smart design achieves the following advantages: (i) the O_2_ in this nanosystem can be precisely released by an NIR-responsive silica shell rupture; (ii) the dynamic biodistribution process of O_2_-PPSiI was monitored in real-time and quantitatively analyzed via sensitive MR imaging of the tumor; (iii) O_2_-PPSiI could alleviate tumor hypoxia by releasing O_2_ within the tumor upon NIR laser excitation; (iv) The migration and invasion abilities of the TNBC tumor were weakened by inhibiting the process of EMT as a result of the synergistic therapy of NIR-triggered O_2_-PPSiI. This NIR responsive on-demand oxygen releasing could provide new thinking on the investigation of controllable drug-releasing nanomedicine systems for precise theranostics in TNBC.

## Supplementary Information

**Additional file 1:** **Figure S1.** (A) The C1s XPS peak of O_2_-PPSi. (B) The C1s XPS peak of O_2_-PPSiI. **Figure S2.** The UV-vis-NIR spectrum of ICG, O_2_-PPSi and O_2_-PPSiI. **Figure S3.** The photographs of O_2_-PPSi and O_2_-PPSiI. **Figure S4.** The magnetic properties of O_2_-PPSiI. **Figure S5.** Normalized absorbance intensity at λ = 808 nm divided by the characteristic length of the cell (A/L) at varied concentrations of O_2_-PPSiI. **Figure S6.** Representative images of PBS and O_2_-PPSiI after irradiated with NIR laser (808 nm, 2 W cm^−2^) for 4 min. **Figure S7.** Temperature changes of ICG and O_2_-PPSiI solution with NIR laser (2 W cm^−2^) switch-on and switch-off for 5 cycles. **Figure S8.** The image of O_2_-PPSiI before and after NIR laser irradiation. The appearance of bubbles suggested the O_2_ release from O_2_-PPSiI after NIR laser irradiation. **Figure S9.** The gray value of ultrasonography for water and O_2_-PPSiI nanosystem solution before and after laser irradiation in vitro. **Figure S10.** The raised temperature of O_2_-PPSiI under NIR laser treatment was the main contributor of oxygen release from O_2_-PPSiI. The scale bar = 100. **Figure S11.** The gray value of ultrasonography for mice before and after treatment with O_2_-PPSiI nanosystem and laser irradiation. **Figure S12.** (A-B) The intracellular uptake of O_2_-PPSil in TNBC cells (MDA-MB-231) and normal breast cells (Hs 578Bst). (C) Effects of RGD and uPA on the intracellular uptake of O_2_-PPSil in MDA-MB-231 cells (MDA-MB-231). (D)The fluorescence imaging of O_2_-PPSiI in vivo. **Figure S13.** The distribution of O_2_-PPSiI in liver, spleen and kidney quantified by T1 mapping, and significant difference between the groups at the same time point is indicated at *P* < 0.05 (*) level. **Figure S14.** Cytotoxicity of O_2_-PPSiI with and without NIR irradiation against MDA-MB-231 cells. **Figure S15.** The overproduction of ^1^O_2_ and ROS induced by NIR-triggered O_2_-PPSiI. **Figure S16.** The separated tumors of each group at the 21 days after the treatment. **Figure S17.** IVIM-DWI derived D mapping of tumor in each groups before and after the treatment, and significant difference of relative D values (△D) between the Saline and treatment groups is indicated at *P* < 0.05 (^*^) or *P* < 0.001 (^**^) level. **Figure S18.** Pearson correlation analysis between MRI-derived parameters and the expression of E-cadherin, vimentin and Snail-Slug, statistically significant correlation coefficient (r) is indicated at *P* < 0.05 (*) or *P* < 0.001 (**) level. **Figure S19.** Pearson correlation analysis between the expression of Snail-Slug/Vimentin and ZEB1, TGF-β and MMP2, statistically significant correlation coefficient (r) is indicated at *P* < 0.05 (*) or *P* < 0.001 (**) level. **Figure S20.** (A) The relative body weight (△Body weight) changes of tumor-bearing mice in each groups at different time points, and (B) the comparison of △Body weight (%) between the Saline and treatment groups at 21 days after treatment. Significant is indicated at *P* < 0.05 (*) or *P* < 0.001 (**) level.

## Data Availability

The datasets used and/or analyzed during the current study are available from the corresponding authors on reasonable request.
