# Supplementary material for: Facile synthesis of near-infrared responsive on-demand oxygen releasing nanoplatform for precise MRI-guided theranostics of hypoxia-induced tumor chemoresistance and metastasis in triple negative breast cancer
Source: J Nanobiotechnology. 2022 Mar 4;20:104. doi: 10.1186/s12951-022-01294-z (PMC8896283; doi:10.1186/s12951-022-01294-z)
Supplement: Supplementary file 1 — Additional file 1: Figure S1. (A) The C1s XPS peak of O2-PPSi. (B) The C1s XPS peak of O2-PPSiI. Figure S2. The UV-vis-NIR spectrum of ICG, O2-PPSi and O2-PPSiI. Figure S3. The photographs of O2-PPSi and O2-PPSiI. Figure S4. The magnetic properties of O2-PPSiI. Figure S5. Normalized absorbance intensity at λ = 808 nm divided by the characteristic length of the cell (A/L) at varied concentrations of O2-PPSiI. Figure S6. Representative images of PBS and O2-PPSiI after irradiated with NIR laser (808 nm, 2 W cm−2) for 4 min. Figure S7. Temperature changes of ICG and O2-PPSiI solution with NIR laser (2 W cm−2) switch-on and switch-off for 5 cycles. Figure S8. The image of O2-PPSiI before and after NIR laser irradiation. The appearance of bubbles suggested the O2 release from O2-PPSiI after NIR laser irradiation. Figure S9. The gray value of ultrasonography for water and O2-PPSiI nanosystem solution before and after laser irradiation in vitro. Figure S10. The raised temperature of O2-PPSiI under NIR laser treatment was the main contributor of oxygen release from O2-PPSiI. The scale bar = 100. Figure S11. The gray value of ultrasonography for mice before and after treatment with O2-PPSiI nanosystem and laser irradiation. Figure S12. (A-B) The intracellular uptake of O2-PPSil in TNBC cells (MDA-MB-231) and normal breast cells (Hs 578Bst). (C) Effects of RGD and uPA on the intracellular uptake of O2-PPSil in MDA-MB-231 cells (MDA-MB-231). (D)The fluorescence imaging of O2-PPSiI in vivo. Figure S13. The distribution of O2-PPSiI in liver, spleen and kidney quantified by T1 mapping, and significant difference between the groups at the same time point is indicated at P < 0.05 (*) level. Figure S14. Cytotoxicity of O2-PPSiI with and without NIR irradiation against MDA-MB-231 cells. Figure S15. The overproduction of 1O2 and ROS induced by NIR-triggered O2-PPSiI. Figure S16. The separated tumors of each group at the 21 days after the treatment. Figure S17. IVIM-DWI derived D mappin [file 12951_2022_1294_MOESM1_ESM.doc]

**Supporting information**

**for**

**Facile synthesis of near-infrared responsive on-demand oxygen releasing nanoplatform for precise MRI-guided theranostics of hypoxia-induced tumor chemoresistance and metastasis in triple negative breast cancer**

^1, 3^Dong Zhang^‡^, ^1,2^Yuanyuan You^‡^, ^1^Yuan Xu, ^1^Qingqing Cheng, ^1^Zeyu Xiao,

^1,2^Tianfeng Chen*, ^1^Changzheng Shi*, ^1^Liangping Luo*

^1^Department of Medical Imaging Center, The First Affiliated Hospital, Jinan University, Guangzhou 510630, China.

^2^ Zhuhai Precision Medical Center, Guangdong Provincial Key Laboratory of Tumor Interventional Diagnosis and Treatment, Zhuhai People's Hospital, Zhuhai Hospital Affiliated with Jinan University, Jinan University, Zhuhai, Guangdong 519000, P.R. China.

^3^The Shunde Affiliated Hospital, Jinan University, Foshan 528300, China.

E-mail: tchentf@jnu.edu.cn, tsczcn@jnu.edu.cn, tluolp@jnu.edu.cn.

^‡^These authors contributed equally to this work.

1. **Experimental Section**

**1.1 Characterization of O_2_-PPSiI**

The morphology of O_2_-PPSiI was observed with transmission electron microscopy (TEM, JEM-2100F, JEOL, Japan). The elemental composition was analyzed with energy-dispersive X-ray spectrometry (EDS, JEOL, Japan) and dark-field scanning TEM (DF-STEM, JEOL, Japan). The UV-vis- NIR spectra test was performed on a UV−vis−NIR spectrophotometer (wavelength ranging from 400 nm to 880 nm). The zeta potential was examined using a Nano-ZS instrument (Malvern Instruments Limited, England).

**1.2 Photothermal Effects of O_2_-PPSiI**

First, the O_2_-PPSiI was irradiated by an 808 nm NIR laser (Laser Technol. Co., Ltd of Xi’an, China) with the laser density set at 0.5, 1.0, 1.5 and 2.0 W/cm^2^. All the temperature data was recorded using an infrared thermograph (Fluke, U.S.).

The O_2_-PPSiI and ICG were irradiated with repeated 808 nm NIR laser switch-on or switch-off for 5 cycles. In each cycle, the O_2_-PPSiI or ICG sample was irradiated by an 808 nm NIR laser at a density of 1.5 W/cm^2^ for 200 s. All the temperature data was recorded using an infrared thermograph (Fluke, U.S.).

**1.3 Magnetic properties of O_2_-PPSiI**

The T1-weighted imaging (T1WI) and T1-mapping MRI of O_2_-PPSiI solutions with different concentrations at 2.25, 4.5, 9, 18 and 36 μM were performed with a 1.5 T Signa HDxt superconductor clinical Magnetic resonance system (GE Medical, Milwaukee, U.S.). The parameters of T1WI were as follows: time of repetition/echo (TR/TE), 340/14.7 ms; field of view (FOV), 100 mm×100 mm; matrix, 192×160; slice thickness/space, 2.0/0.2 mm. The parameters of T1-mapping MRI were as follows: TR/TE, 4000/12.3 ms; FOV 100 mm×100 mm; matrix, 224×128; slice thickness/space, 2.0/0.2 mm; time of inversion (TI), 100, 200, 300, 400, 500 and 600 ms. All the T1-mapping images were transferred to a dedicated postprocessing workstation (AW4.5, GE Healthcare) and the T1 value of each sample was measured, and then the T1 relaxivity (r1) was obtained through linear fitting calculation.

**1.4 Cell Culture and Cytotoxicity Assay *In Vitro***

All the cell lines used in this study were purchased from American Type Culture Collection (ATCC, Manassas, Virginia, USA), including the MDA-MB-231 (human triple-negative breast cancer cell line) and HS578Bst (normal breast epithelial cell line). Both the cell lines were incubated in Dulbecco’s modified Eagle’s medium (Gibco, Paisley, United Kingdom) with the supplements of 50 u/mL streptomycin (Sigma-Aldrich), 100 u/mL penicillin (Sigma Aldrich) and 10% fetal bovine serum (Gibco) at 37℃ in a 5% CO_2_ incubator.

The thiazolyl blue tetrazolium bromide (MTT) assay was performed to investigate the cytotoxicity of O_2_-PPSiI. MDA-MB-231 and HS578Bst cells were seeded into a 96-well plate (2×10^4^ cells/mL, 100 μL/well) and cultured for 24 h in the medium. And then, the cells were treated with different groups at equivalent concentrations of 0.25 μg/mL PTx. After incubation for 24 h, 30 μL/well of MTT solution was added and incubated for 3 h. In the end, the medium was replaced with 150μL/well dimethyl sulfoxide (DMSO) and dissolved for 10 min, and then the absorbance at 570 nm was recorded with a microplate spectrophotometer (SpectroA maxTM250).

**1.5 Measurement of ROS and ^1^O_2_ Generation**

The measurement of intracellular ROS and ^1^O_2_ generation was performed with DHE and DPBF fluorescence assays. Briefly, the MDA-MB-231 cells were stained with 10 μM DHE or DPBF at 37℃ for 30 min, and then exposed to different treatment groups at equivalent concentrations of 0.25 μg/mL PTx and/or NIR laser (808 nm, 2 W cm^-2^, 2min) Next, the fluorescence intensities of ROS and ^1^O_2_ were monitored with a fluorescence microplate reader.

**1.6 MR imaging of O_2_-PPSiI *in Vivo***

To investigate the distribution of O_2_-PPSiI in vivo, the included tumor-bearing mice were divided into 2 groups. The O_2_-PPSiI and free Gd-DTPA were injected through the caudal vein at equivalent concentration of 0.03 mmol/kg Gd-DTPA. MR imaging including T1WI, T2WI and T1 mapping sequences was performed with a 1.5 T Signa HDxt superconductor clinical Magnetic resonance system (GE Medical, Milwaukee, U.S.) before and after the administration of 30 min, 2 h, 4 h, 8 h, 12 h, 24 h, 48 h, 72 h, and 96 h. As the free Gd-DTPA was soon metabolized from the body, the MR imaging of Gd-DTPA group was only monitored within 8 h. All the T1-mapping images were transferred to a dedicated postprocessing workstation (AW4.5, GE Healthcare) and the T1 values of tumor, liver, spleen and kidney were measured, and its percentage difference was calculated as △T1 (%) = (T1*^i^*-T1*^base^*) /T1*^base^*×100%, which *i* represented the different time points. The parameters of T1WI were as follows: TR/TE, 340/14.7 ms; FOV 50 mm×50 mm; matrix, 192×160; slice thickness/space, 2.0/0.2 mm. The parameters of T2WI were as follows: TR/TE, 1900/82.3 ms; FOV 50 mm×50 mm; matrix, 192×160; slice thickness/space, 2.0/0.2 mm. T1 mapping of the tumor was performed with multiple TI acquisition: TR/TE, 4000/12.3 ms; FOV 50 mm×50 mm; matrix, 224×128; slice thickness/space, 2.0/0.2 mm; time of inversion (TI), 100, 200, 300, 400, 500 and 600 ms. As for the liver, spleen and kidney, the T1 mapping was performed with multiple flip angle (FA) acquisition: TR/TE, 40/2.4 ms; FOV 50 mm×50 mm; matrix, 128×96; slice thickness/space, 2.0/0.2 mm; FA, 3°, 6°, 9°, 12° and 15°. Besides, by capturing the fluorescence signal of ICG, the fluorescence imaging was also performed using a live imaging system (IVIS, Xenogen, USA).

To further investigate the penetration of O_2_-PPSiI in the tumor, a perfusion-based segmentation and region-of-interest (ROI) analysis were performed with functional MRI. Briefly, in the first stage, seven tumor-bearing mice were selected randomly, and was scanned with intravoxel incoherent motion diffusion-weighted imaging (IVIM-DWI) sequence before the administration with TR/TE, 3000/101.7 ms; FOV 50 mm×50 mm; matrix, 128×96; slice thickness/space, 2.0/0.2 mm; b values, 0, 25, 50, 75, 100, 150, 200, 400, 600, 800, 1000, 1200 and 1500 s/mm^2^; and then the perfusion-related parameter f was mapped via the Functool MADC program at the post-processing workstation (AW4.5, GE Healthcare) [1]. Next, the largest cross-sectional image of f mapping was selected, and the f value was measured as a function of distance from the tumor edge to center. Two orthogonal lines in the Ventral/Dorsal (Ve/Do) and Lateral/Medial (La/Me) directions were drawn cross the tumor center, and divided into four radii. Each radius was further divided into 10 segments with equivalent length, and each segment was then assigned with a value of 1 – 10 (i.e., the outermost segment was assigned with 1 and the innermost segment a radial position of 10). Because the orthogonal lines were nearly symmetric, the obtained data of two symmetrical radii were pooled and the radial heterogeneity in perfusion was statistically analyzed. Finally, a favorable proportion of segments with significantly decreased perfusion compared to the edge (segment 1) in both the Ve/Do and La/Me directions was investigated. Thereafter, the ROI of tumor center and periphery was defined and the penetration of administrated O_2_-PPSiI in the tumor was investigated by △T1 (%) as descripted above.

**1.7 Histological Analysis**

All the tumors and organs excised were fixed in 4% paraformaldehyde solutions and embedded in the paraffin. And then the samples were sliced with 5 mm thickness. Afterwards, the slices were stained with H&E. Besides, the HIF-1α, Vimentin, Snail-Slug and E-cadherin staining were also performed and observed with fluorescence microscope or optical inverted microscope. The further analysis was performed with “hot spots” methods. Briefly, three most positive areas were captured at the magnification of 10×20, and then the integrated optical density (IOD) of positive cells on each image was calculated using Image-Pro Plus 6.0 software (Media Cybernetics).

**1.8 Biosafety Evaluation**

The blood was sampled from the eyeballs of the mice with 0.2-0.3 mL, and then was centrifuged for 100-150 μL. All the samples were analyzed with a Hitachi 7600 biochemical analyzer, and the indexes of liver, renal and heart function was tested, including urea nitrogen (BUN), creatinine (CREA), Lactate dehydrogenase (LDH) Creatine kinase (CK), Alanine transaminase (ALT), Aspartate aminotransferase (AST), Albumin (ALB), Globulin (GLB) and Total protein (TP).

**2. Data Section**


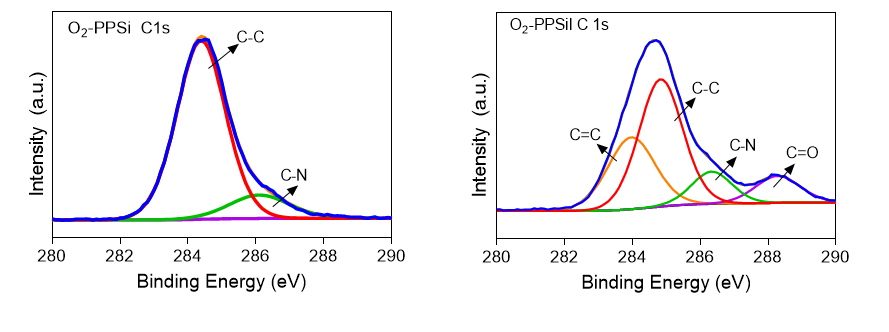


**Figure S1**. (A) The C1s XPS peak of O_2_-PPSi. (B) The C1s XPS peak of O_2_-PPSiI.

**Figure S2**. The UV-vis-NIR spectrum of ICG, O_2_-PPSi and O_2_-PPSiI.


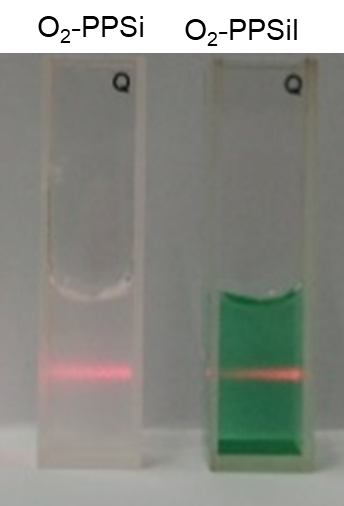


**Figure S3**. The photographs of O_2_-PPSi and O_2_-PPSiI.


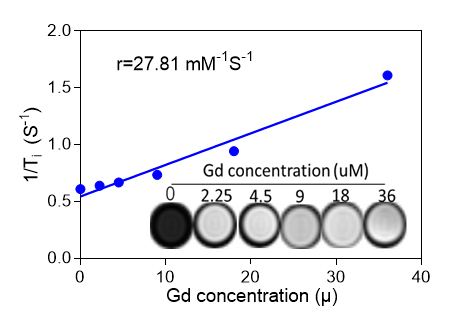


**Figure S4**. The magnetic properties of O_2_-PPSiI.

**Figure S5**. Normalized absorbance intensity at λ = 808 nm divided by the characteristic length of the cell (A/L) at varied concentrations of O_2_-PPSiI.


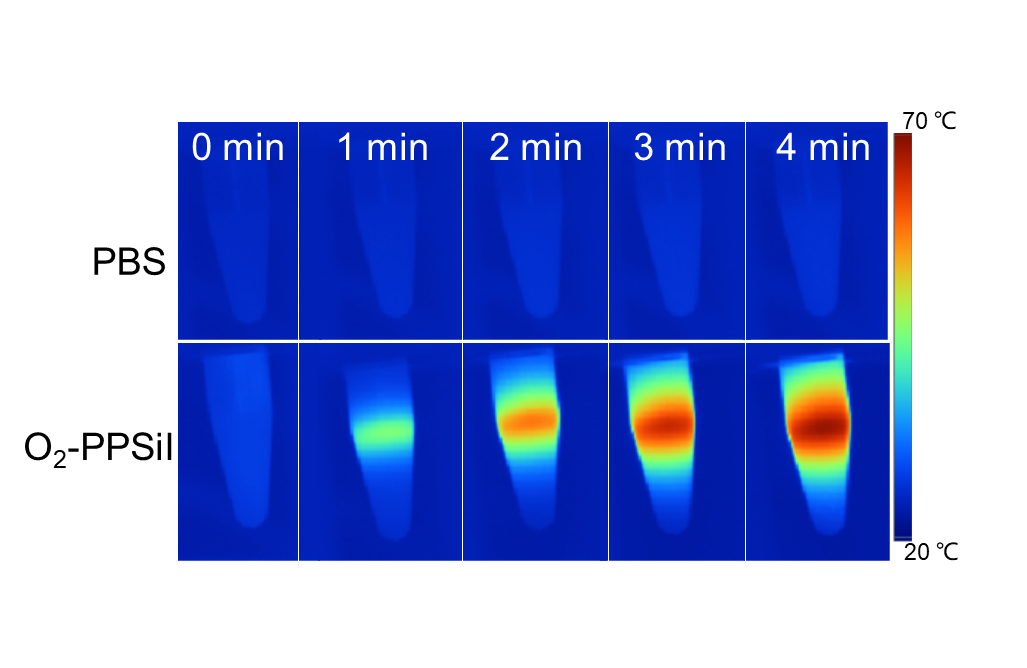


**Figure S6**. Representative images of PBS and O_2_-PPSiI after irradiated with NIR laser (808 nm, 2 W cm^−2^) for 4 min.

**Figure S7**. Temperature changes of ICG and O_2_-PPSiI solution with NIR laser (2 W cm^−2^) switch-on and switch-off for 5 cycles.


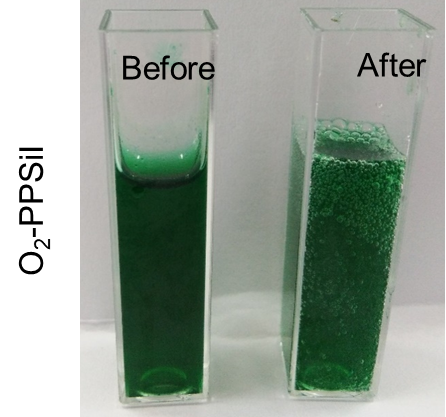


**Figure S8**. The image of O_2_-PPSiI before and after NIR laser irradiation. The appearance of bubbles suggested the O_2_ release from O_2_-PPSiI after NIR laser irradiation.

**Figure S9**. The gray value of ultrasonography for water and O_2_-PPSiI nanosystem solution before and after laser irradiation *in vitro*.


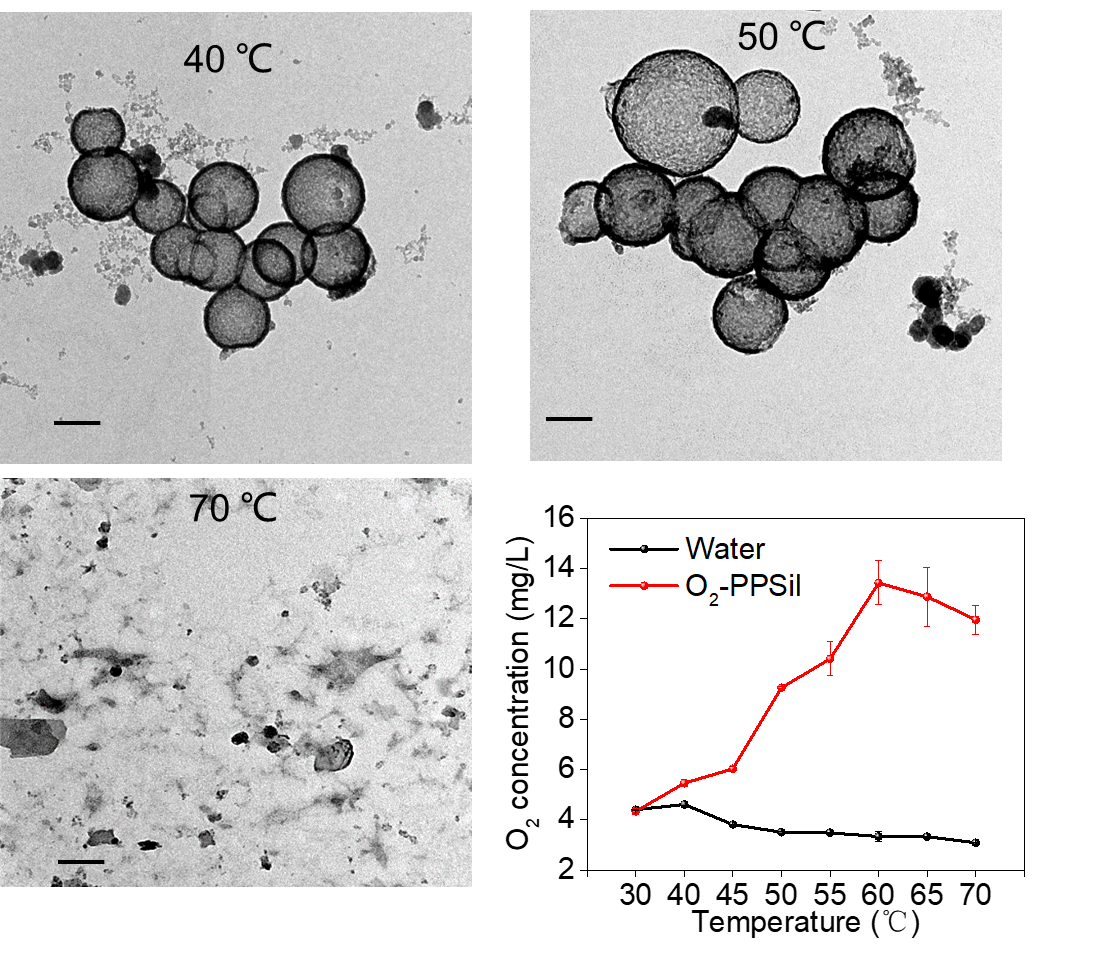


**Figure S10**. The raised temperature of O_2_-PPSiI under NIR laser treatment was the main contributor of oxygen release from O_2_-PPSiI. The scale bar = 100 nm.

**Figure S11**. The gray value of ultrasonography for mice before and after treatment with O_2_-PPSiI nanosystem and laser irradiation.


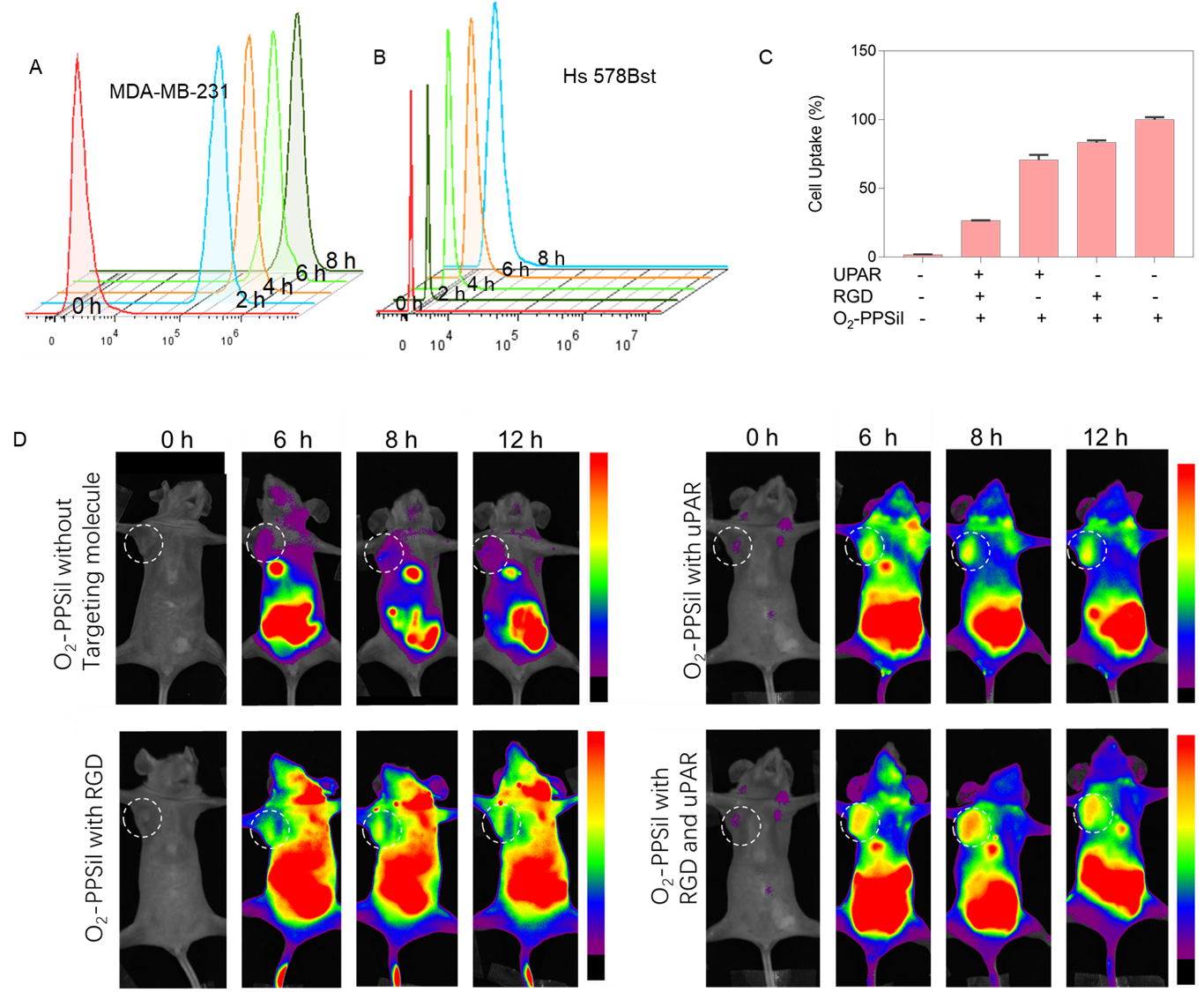


**Figure S12**. (A-B) The intracellular uptake of O_2_-PPSil in TNBC cells (MDA-MB-231) and normal breast cells (Hs 578Bst). (C) Effects of RGD and uPA on the intracellular uptake of O_2_-PPSil in MDA-MB-231 cells (MDA-MB-231). (D)The fluorescence imaging of O_2_-PPSiI *in vivo*.


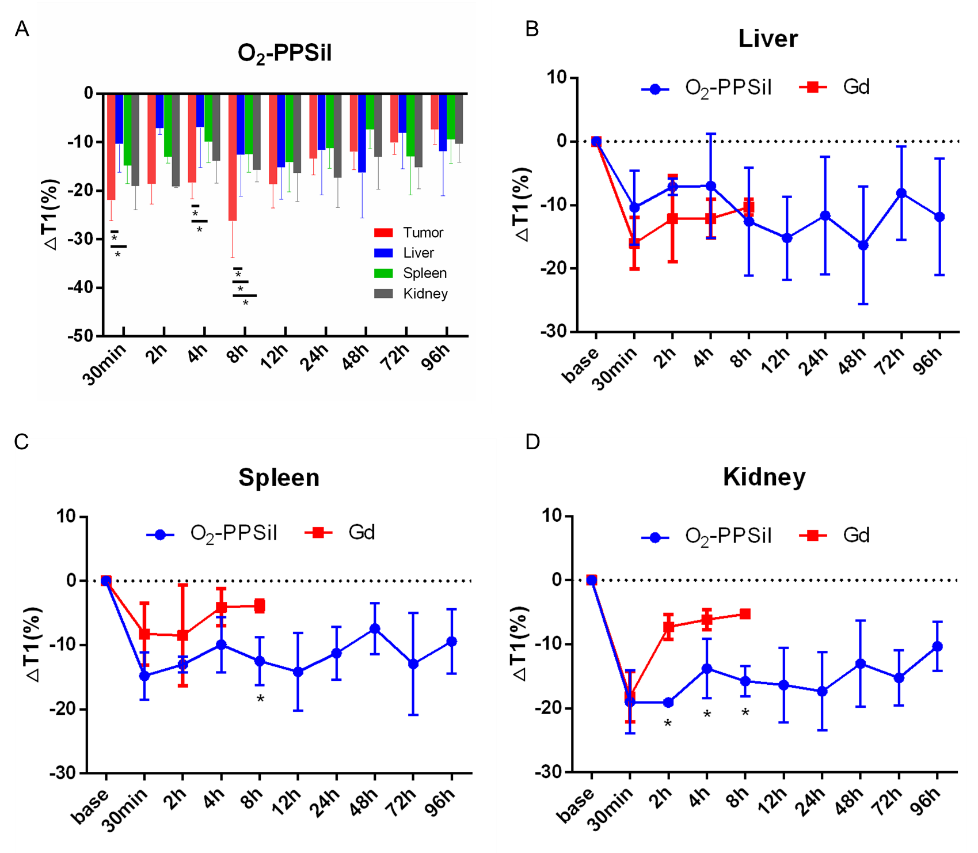


**Figure S13**. The distribution of O_2_-PPSiI in liver, spleen and kidney quantified by T1 mapping, and significant difference between the groups at the same time point is indicated at *P* < 0.05 (*) level.


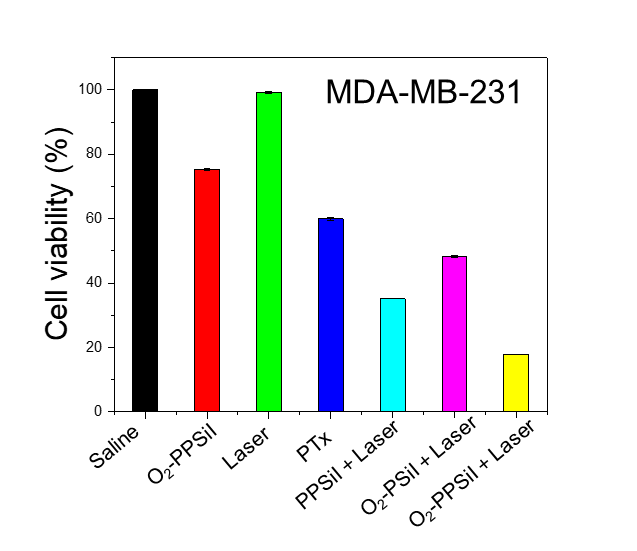


**Figure S14**. Cytotoxicity of O_2_-PPSiI with and without NIR irradiation against MDA-MB-231 cells.


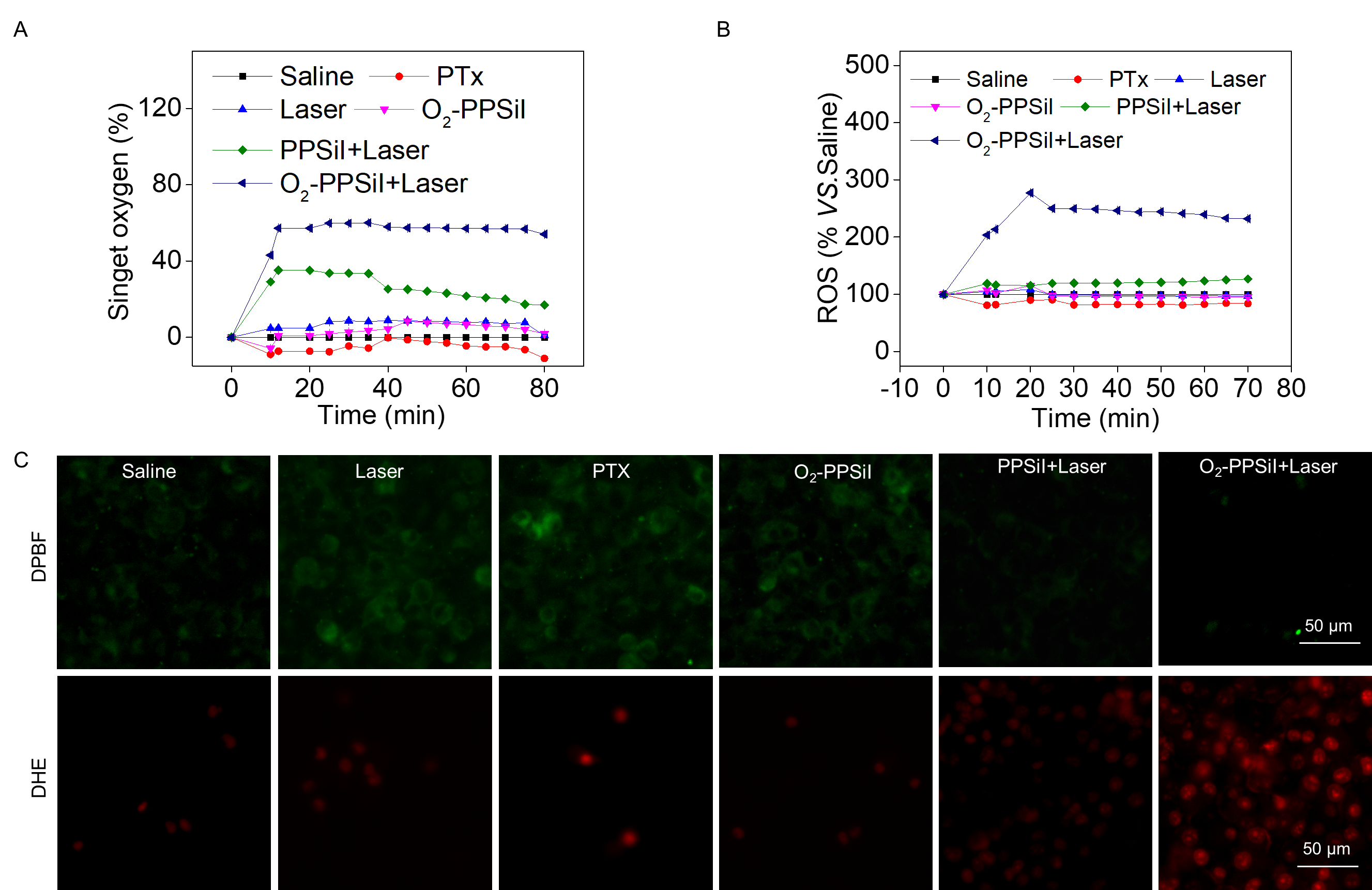


**Figure S15**. The overproduction of ^1^O_2_ and ROS induced by NIR-triggered O_2_-PPSiI.


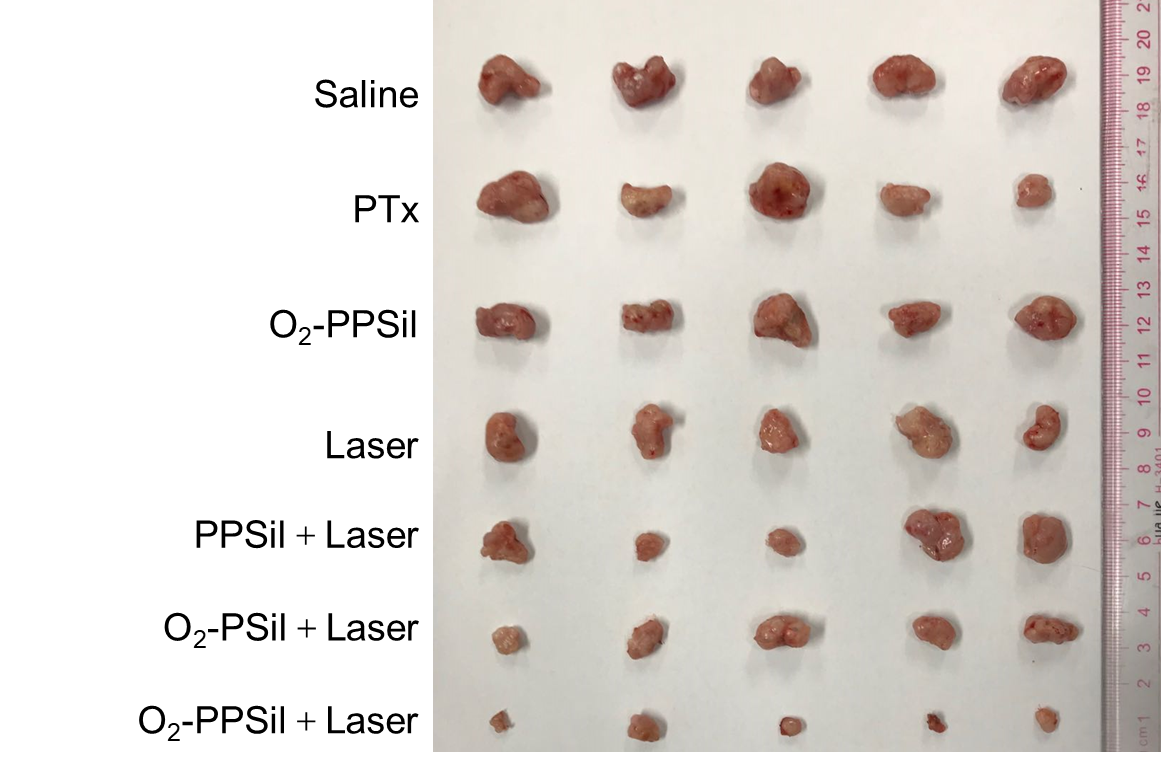


**Figure S16**. The separated tumors of each group at the 21 days after the treatment.


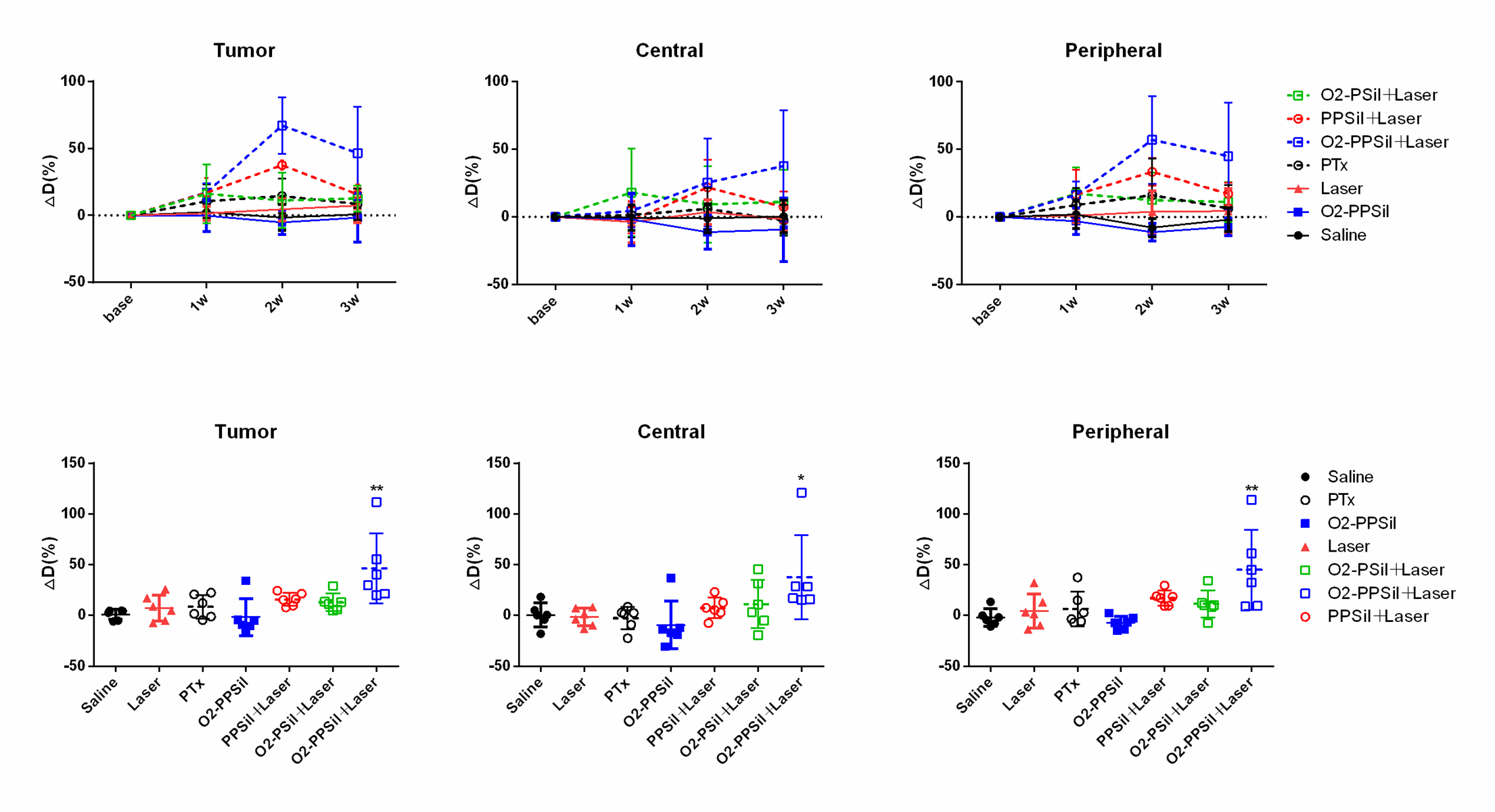


**Figure S17**. IVIM-DWI derived D mapping of tumor in each groups before and after the treatment, and significant difference of relative D values (△D) between the Saline and treatment groups is indicated at *P* < 0.05 (^*^) or *P* < 0.001 (^**^) level.


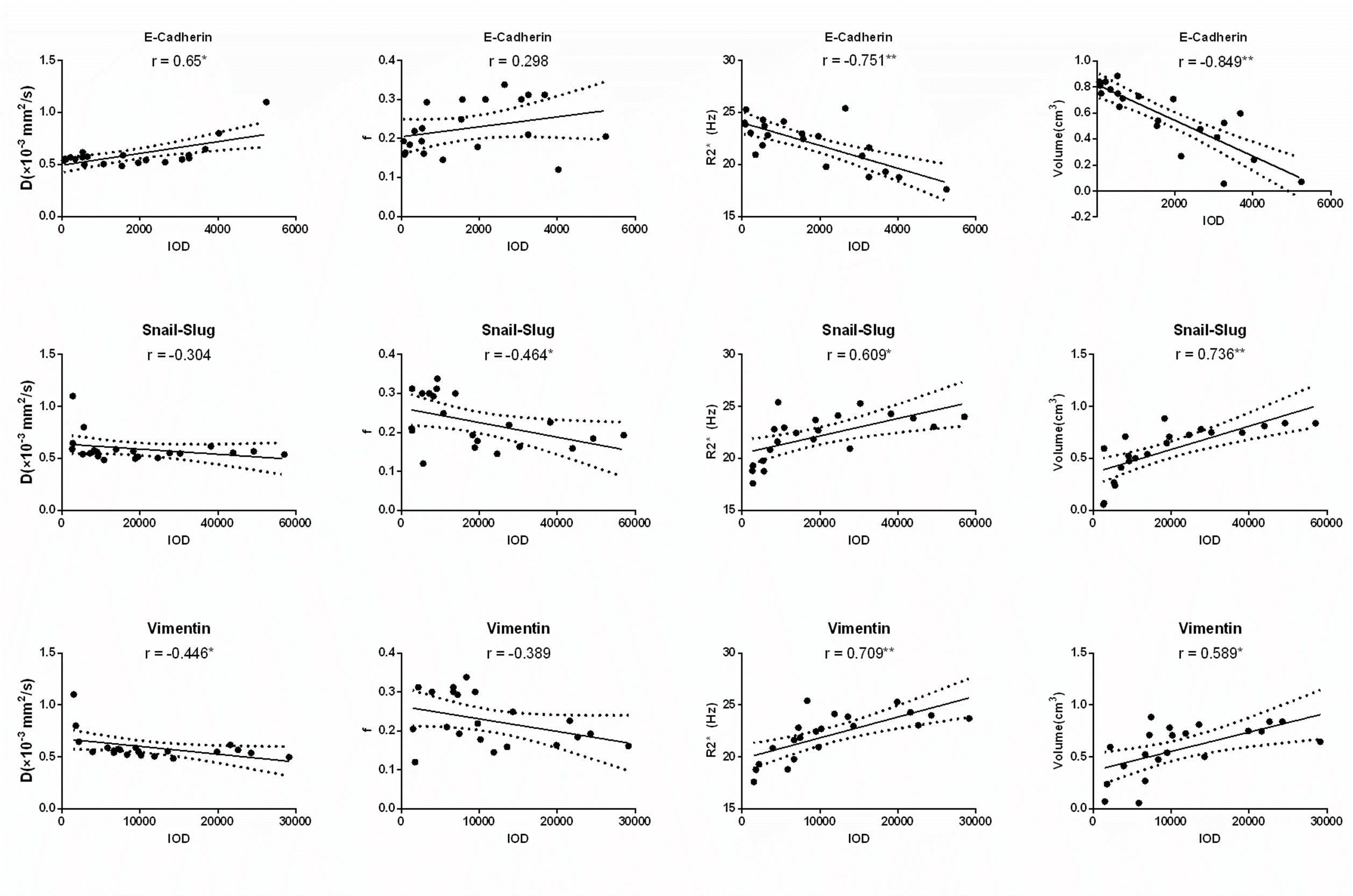


**Figure S18**.Pearson correlation analysis between MRI-derived parameters and the expression of E-cadherin, vimentin and Snail-Slug, statistically significant correlation coefficient (r) is indicated at *P* < 0.05 (*) or *P* < 0.001 (**) level.


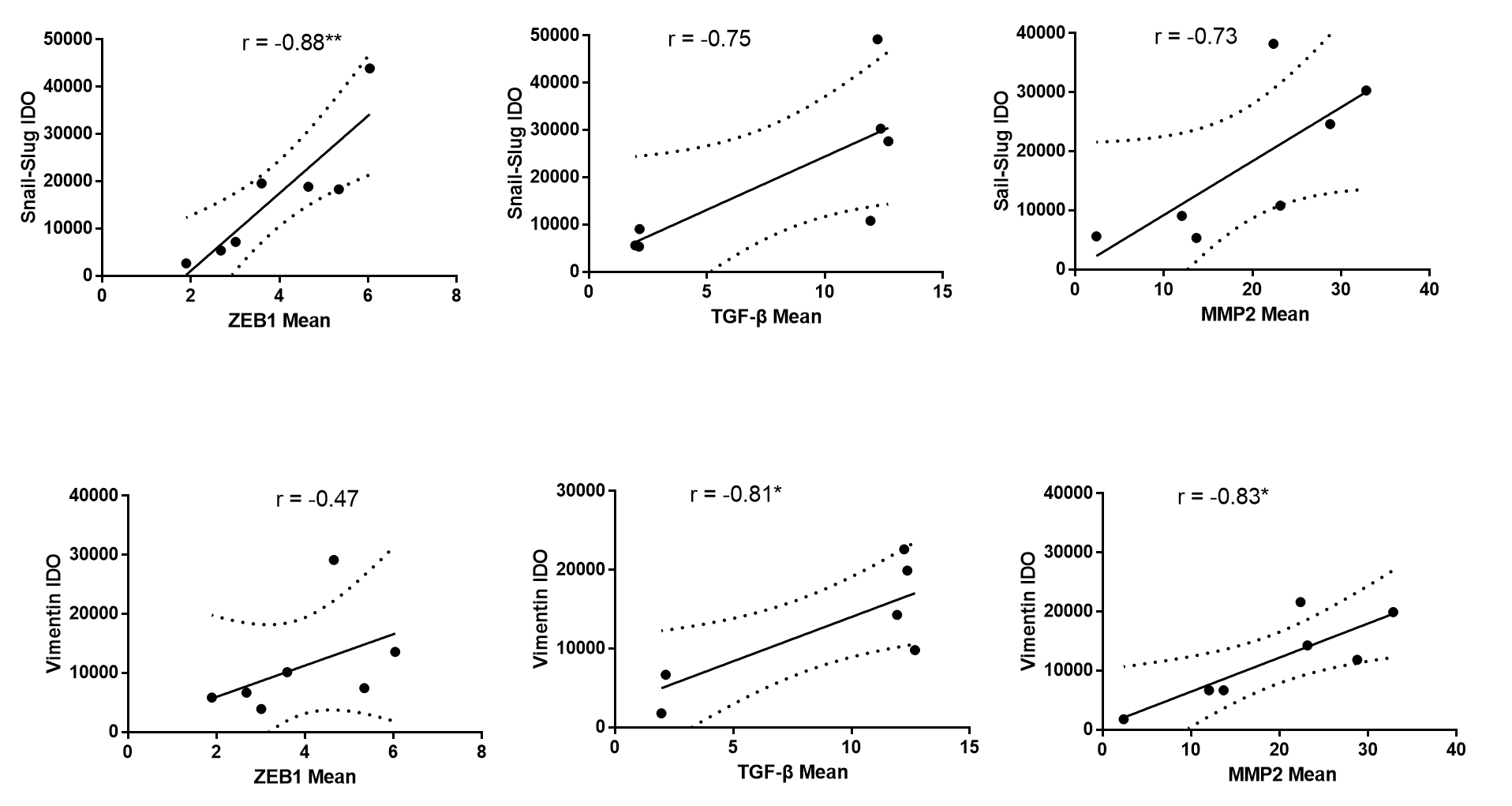


**Figure S19.** Pearson correlation analysis between the expression of Snail-Slug/Vimentin and ZEB1, TGF-β and MMP2, statistically significant correlation coefficient (r) is indicated at *P* < 0.05 (*) or *P* < 0.001 (**) level.

**
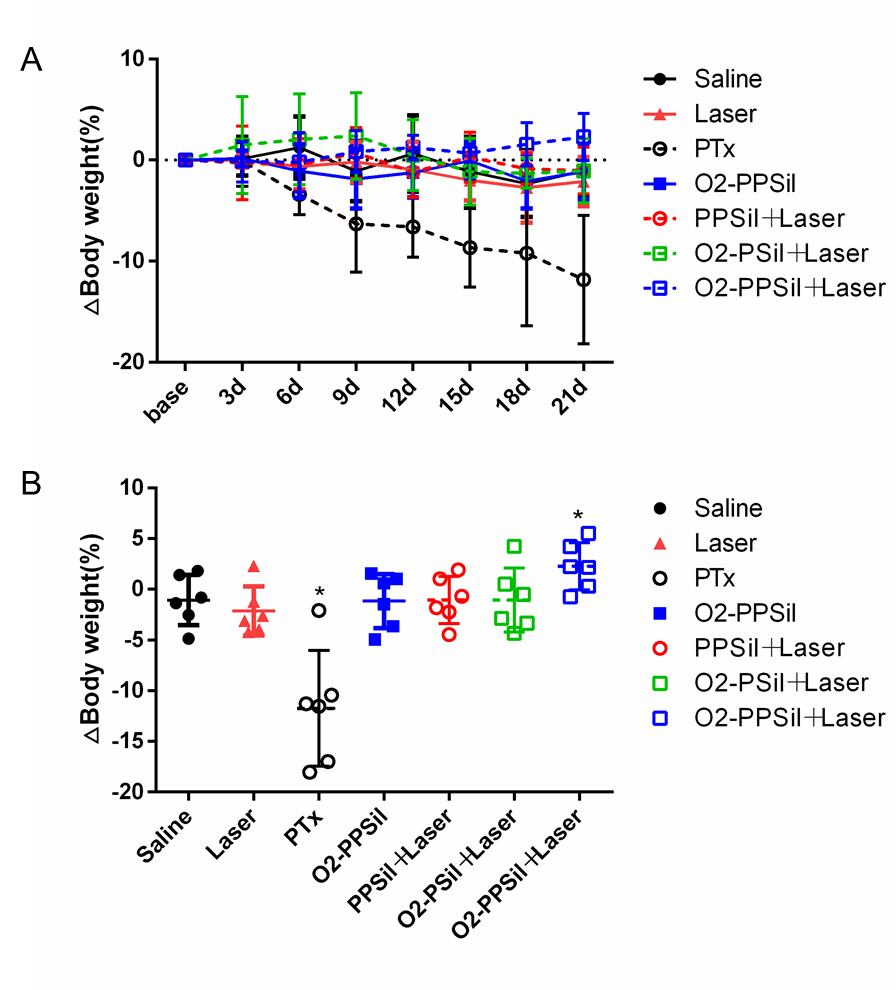
**

**Figure S20.** (A) The relative body weight (△Body weight) changes of tumor-bearing mice in each groups at different time points, and (B) the comparison of △Body weight(%) between the Saline and treatment groups at 21 days after treatment. Significant is indicated at *P* < 0.05 (*) or *P* < 0.001 (**) level.
